# Confirmation of Childhood Acute Lymphoblastic Leukemia Variants, *ARID5B* and *IKZF1*, and Interaction with Parental Environmental Exposures

**DOI:** 10.1371/journal.pone.0110255

**Published:** 2014-10-13

**Authors:** Tiffany-Jane Evans, Elizabeth Milne, Denise Anderson, Nicholas H. de Klerk, Sarra E. Jamieson, Bente A. Talseth-Palmer, Nikola A. Bowden, Elizabeth G. Holliday, Jérémie Rudant, Laurent Orsi, Ebony Richardson, Laura Lavis, Daniel Catchpoole, John R. Attia, Bruce K. Armstrong, Jacqueline Clavel, Rodney J. Scott

**Affiliations:** 1 Centre for Bioinformatics, Biomarker Discovery and Information-Based Medicine, Hunter Medical Research Institute, Newcastle, New South Wales, Australia; 2 School of Biomedical Sciences and Pharmacy, University of Newcastle, Newcastle, New South Wales, Australia; 3 Telethon Kids Institute, The University of Western Australia, Perth, Western Australia, Australia; 4 Centre for Clinical Epidemiology and Biostatistics, School of Medicine and Public Health, University of Newcastle, Newcastle, New South Wales, Australia; 5 INSERM, U1018, Department of Environmental Epidemiology of Cancers, Villejuif, France; 6 Paris-Sud University, UMR-S1018, Department of Environmental Epidemiology of Cancers, Research Center in Epidemiology and Population Health, Villejuif, France; 7 French National Registry of Childhood Blood Malignancies (RNHE), Villejuif, France; 8 The Tumour Bank, The Children’s Cancer Research Unit, The Children’s Hospital Westmead, Westmead, New South Wales, Australia; 9 Department of Medicine, John Hunter Hospital and Hunter Medical Research Institute, New Lambton, New South Wales, Australia; 10 School of Public Health, The University of Sydney and Sax Institute, Sydney, New South Wales, Australia; 11 Molecular Genetics, Hunter Area Pathology Service, Newcastle, New South Wales, Australia; National Health Research Institutes, Taiwan

## Abstract

Genome wide association studies (GWAS) have established association of *ARID5B* and *IKZF1* variants with childhood acute lymphoblastic leukemia (ALL). Epidemiological studies suggest that environmental factors alone appear to make a relatively minor contribution to disease risk. The polygenic nature of childhood ALL predisposition together with the timing of environmental triggers may hold vital clues for disease etiology. This study presents results from an Australian GWAS of childhood ALL cases (n = 358) and population controls (n = 1192). Furthermore, we utilised family trio (n = 204) genotypes to extend our investigation to gene-environment interaction of significant loci with parental exposures before conception, and child’s sex and age. Thirteen SNPs achieved genome wide significance in the population based case/control analysis; ten annotated to *ARID5B* and three to *IKZF1*. The most significant SNPs in these regions were *ARID5B* rs4245595 (OR 1.63, CI 1.38–1.93, *P* = 2.13×10^−9^), and *IKZF1* rs1110701 (OR 1.69, CI 1.42–2.02, p = 7.26×10^−9^). There was evidence of gene-environment interaction for risk genotype at *IKZF1*, whereby an apparently stronger genetic effect was observed if the mother took folic acid or if the father did not smoke prior to pregnancy (respective interaction P-values: 0.04, 0.05). There were no interactions of risk genotypes with age or sex (P-values >0.2). Our results evidence that interaction of genetic variants and environmental exposures may further alter risk of childhood ALL however, investigation in a larger population is required. If interaction of folic acid supplementation and *IKZF1* variants holds, it may be useful to quantify folate levels prior to initiating use of folic acid supplements.

## Introduction

Contemporary genome wide association studies (GWAS) have consistently revealed, in the European population, two genetic loci that are associated with childhood acute lymphoblastic leukemia (ALL) risk [1]–[4]. The genes implicated at these loci, *AT-rich interactive domain 5b* (*ARID5B*) and *Ikaros family zinc finger 1* (*IKZF1*), encode proteins that regulate normal lymphocyte differentiation. Other genes have been identified from some but not all GWASs including *CEBPE, CDKN2A* and *GATA3* [5], [6]. A recent report [7] demonstrated a greater sensitivity to detect risk loci by using an ethnically diverse population of childhood ALL patients and identified a new risk locus at 10p12.31 (*BMI1-PIP4K2A*). Also, a graduated scale of ALL risk was demonstrated for: i) increasing number of risk alleles at four of the loci combined (*ARID5A, IKZF1, CEBPE* and *BMI1-PIP4K2A*); and ii) an *ARID5B* risk allele with odds highest for children younger than five years and lowest for those older than 10 years. This information underscores the polygenic nature of childhood ALL and places some emphasis on the potential for interaction with environmental triggers and the developmental stages at which they occur.

Epidemiological studies have assessed the role of environmental factors in the pathogenesis of childhood ALL however these factors alone appear to make a relatively minor contribution to disease risk [8]. Previous investigations from our own Australian ALL case-control study have focussed on parental exposures before and after conception including: alcohol consumption, folic acid use, and smoking. We found that paternal smoking around time of conception increased the odds of childhood ALL [9]; our meta-analysis for maternal folate demonstrated that supplementation had a protective effect [10] and; while parental alcohol consumption did not alter odds, the quantity of consumption might [11].

Aforementioned GWAS have investigated associations of *IKZF1* and *ARID5B* genotypes with treatment outcomes, and Linabery *et al*. [12] assessed interaction with age and sex demographics; however, we hypothesise that genetic variants compounded with environmental exposures and the developmental stages at which they occur, may contribute to the background of risk in childhood ALL.

This study presents results from an Australian GWAS of childhood ALL cases and population controls. A French case-control study was used to determine the validity of novel findings [3]. Finally, we investigated gene-environment interaction of key loci identified in our GWAS with parental exposures before conception, and child’s sex and age.

## Materials and Methods

### Study population

The study population comprised 441 childhood ALL cases from whom blood-derived DNA had been isolated during remission. 324 were participants in The Australian Study of Childhood Acute Lymphoblastic Leukaemia (Aus-ALL) described in Milne et al. [10]. This study involved the collection of environmental exposure data via questionnaire. The remaining 117 cases were from The Tumour Bank at the Children’s Hospital Westmead in Sydney NSW, consecutively collected from 1998–2006. The control series comprised 1229 healthy Caucasian adults sourced from the Hunter Community Study (HCS), Newcastle, Australia [13]. Aus-ALL was approved by the human research ethics committees at all participating hospitals and the Hunter Community study was approved by the University of Newcastle Research Ethics Committee and the Hunter New England Health Service Research Ethics Committee.

### Genome-wide genotyping

Genome-wide genotyping was undertaken on Illumina (San Diego, USA) BeadChips accordingly: 102 cases were genotyped for the pilot study using HumanCNV370-Duo v.1; 191 cases using HumanCNV370-Quad v.3; 148 cases and 1229 controls using Human610-Quad v.1. Genotype validation for rs7089424 and rs4132601 was carried out for the Aus-ALL cases and 400 HCS controls using Applied Biosystems (Foster City, USA) TaqMan Technology, revealing 100% concordance. Genotyping for these single nucleotide polymorphisms (SNPs) was extended to both parents of Aus-ALL cases for whom DNA and exposure data were available (n = 204 trios).

### Genome-wide association analysis

PLINK v1.07 [14] was used to apply stringent quality control (QC) first by BeadChip version and again following data merging (Methods S1 in File S1) resulting in 358 cases, 1192 controls and 309 117 SNPs carried forward to genotype imputation (Post-QC case demographics for age, gender and ALL subtype available in Table S1 in File S1). Samples were pre-phased using SHAPEIT, and imputation using the 1000 Genomes phase 1 reference panel was carried out using IMPUTE2 v2.3.0. Case-control association at each SNP was tested under an additive model using a missing data likelihood score test which takes into account genotype uncertainty due to imputation. Models were fitted using SNPTEST v2.4.1, adjusting for the top three principal components defining ancestry. SNPs with a SNPTEST information measure less than 0.4 and a minor allele frequency less than 2% (in cases or controls or cases/controls combined) were filtered out resulting in 7 162 141 SNPs passing QC. Regional association plots for significant loci were produced using LocusZoom [15]. Pritchard Lab and Blood eQTL browsers [16], [17] were used to establish whether SNPs are expression quantitative trait loci (eQTLs).

Data from the French ESCALE nationwide registry, comprising 441 cases and 1542 controls [3], were available for replication of a new suggestive association.

### Gene-environment interaction analyses

For genotyped SNPs at genome-wide significant loci, the method of Cordell [18] was used to investigate gene-environment interactions for 204 cases-parent trios. Interactions with paternal smoking before conception, and maternal use of folic acid prior to pregnancy were investigated because they demonstrated main effects in previous Aus-ALL studies [9], [10]. Maternal alcohol consumption before pregnancy was also investigated as adequate numbers in each exposure category was achievable [11]. Interactions with the child’s sex and age were also assessed. The Stata function pseudocc [19] coupled with conditional logistic regression was used for analysis. Genotype-based (rather than allele based) odds ratios were estimated as recommended by Sasieni [20]. This process uses the parents’ genotypes to estimate the theoretical genotypes their offspring could have had, and these are used as matched controls for each case. Thus, only genetic main effects and their interactions with ‘environmental’ (age, sex, folate, etc) effects can be estimated as each case/control set has the same values for the environmental variables.

## Results

### Genome-wide association study for childhood acute lymophoblastic leukemia

QQ-plot and genomic inflation factor (λ = 1.0006) indicated an absence of population substructure or other systematic bias between cases and controls (Figure S1 in File S1).

Table 1 annotates 13 SNPs at two loci that were significantly associated with childhood ALL (*P*<5×10^−8^), and 259 additional SNPs achieved *P*<10^−5^ (Table S2 in File S1 and Manhattan plot in Figure S2 in File S1). Ten of the 13 significant SNPs map to intron three of *ARID5B* on chromosome 10 and three flank *IKZF1* on chromosome 7 (LocusZoom plots in Figure S3 in File S1).

**Table 1:** SNPs associated with Childhood ALL at *P<*5×10^−8^.

					Alleles(A/B)^b^	ALL v Controls				B-ALL (n = 319) vs Controls (n = 1192)	
						MAF					
Typed^a^	rs ID	Chr	Position	Gene (location)		Cases (n = 358)	Controls (n = 1192)	Allelic OR(95% CI)^c^	P	Allelic OR(95% CI)^c^	P
---	rs4245595	10	63722895	*ARID5B (intronic)*	T/C	0.46	0.34	1.63 (1.38–1.93)	2.13E-09	1.86 (1.54–2.25)	1.21E-10
---	rs7090445	10	63721176	*ARID5B (intronic)*	T/C	0.46	0.34	1.63 (1.37–1.93)	2.81E-09	1.83 (1.51–2.20)	2.88E-10
---	rs4948492	10	63719739	*ARID5B (intronic)*	T/C	0.46	0.34	1.63 (1.37–1.93)	2.84E-09	1.82 (1.51–2.20)	2.91E-10
---	rs7896246	10	63724390	*ARID5B (intronic)*	G/A	0.46	0.34	1.61 (1.36–1.91)	4.63E-09	1.81 (1.50–2.19)	5.11E-10
---	rs4245597	10	63725942	*ARID5B (intronic)*	A/G	0.46	0.35	1.6 (1.35–1.9)	5.87E-09	1.81 (1.50–2.19)	5.99E-10
---	rs10821937	10	63723909	*ARID5B (intronic)*	T/C	0.46	0.35	1.6 (1.35–1.9)	6.36E-09	1.81 (1.50–2.18)	6.96E-10
---	rs10821936	10	63723577	*ARID5B (intronic)*	T/C	0.46	0.35	1.6 (1.35–1.9)	6.36E-09	1.81 (1.50–2.18)	6.95E-10
---	rs7087507	10	63745689	*ARID5B (intronic)*	A/G	0.46	0.35	1.62 (1.37–1.92)	6.96E-09	1.80 (1.50–2.18)	6.99E-10
---	rs1110701	7	50478627	*IKZF1 (3′ Flanking)*	A/G	0.39	0.28	1.69 (1.42–2.02)	7.26E-09	1.91 (1.57–2.32)	8.27E-11
---	rs9415635	10	63753482	*ARID5B (intronic)*	A/G	0.46	0.34	1.6 (1.35–1.89)	2.17E-08	1.76 (1.46–2.12)	2.96E-09
rs7089424	rs7089424	10	63752159	*ARID5B (intronic)*	T/G	0.46	0.34	1.6 (1.35–1.89)	2.35E-08	1.76 (1.46–2.12)	3.26E-09
---	rs10272724	7	50477213	*IKZF1 (3′ Flanking)*	T/C	0.37	0.26	1.67 (1.4–1.99)	3.71E-08	1.90 (1.56–2.31)	1.93E-10
---	rs17133807	7	50477687	*IKZF1 (3′ Flanking)*	G/A	0.37	0.26	1.66 (1.39–1.98)	3.88E-08	1.90 (1.56–2.32)	1.93E-10

a Genotyped SNPs indicated in this column, dashed entries indicate imputed SNPs.

b Allele B is the minor allele for all SNPs in this table.

c Odds ratios represent the effect of additional copies of the B (minor allele).

Abbreviations: MAF, Minor Allele Frequency; Chr, Chromosome; OR, Odds Ratio; CI, Confidence Interval; P, P-value; B-ALL, B-cell Acute Lymphoblastic Leukemia.

The most significantly associated SNP was rs4245595 at *ARID5B* (OR 1.63, CI 1.38–1.93, *P* = 2.13×10^−9^) where G is the risk allele, and the association was stronger for B-cell ALL (OR 1.86, CI 1.54–2.25, *P* = 1.21×10^−10^).

Association at the *IKZF1* locus was most significant for rs1110701 (OR 1.69, CI 1.42–2.02, p = 7.26×10^−9^) where G is the risk allele and this association was also stronger when analysis was confined to the B-cell subtype (OR 1.91, CI 1.57–2.32, *P* = 8.27×10^−11^).

For the third most significant genotyped loci, located on chromosome 8 in the vicinity of the *ZNF704* gene, borderline significance was achieved (*P*<10^−5^). There were three genotyped SNPs in the region with 10^−8^<*P*<10^−5^: rs7000234, rs7018449 and rs6992620. This result, however, was not replicated when interrogated in the French dataset (Table S3 in File S1 and Figure S4 in File S1).

### Interaction analyses of environmental variables and IKZF1 and ARID5B variants

Main effects of genotyped SNPs at *ARID5B* and *IKZF1* loci (rs7089424 and rs4132601 respectively) and interactions with environmental variables were conducted using a reduced population of 204 case-parent trios for whom environmental exposure data were available. Genetic main effects demonstrated significant risk increases with homozygosity for the minor alleles (GG for both) versus the referent homozygous major alleles (TT) (Table 2 and Table S4 in File S1).

**Table 2:** Estimates for genetic main effects of variation at rs4132601 and interaction with parental exposures, age and sex for 204 case-trios.

				Genotype-wise Association			
Variable Name	Genotype	P	Environmental Exposure Present	Cases: Controls^a^	OR^b^	95% CI	P
Restricted to non-missing questionnaires							
**rs4132601**	(GG)			25∶41∶00	2.53	1.31–4.91	**0.006**
	(GT)	**0.02** c	None	84∶260	1.2	0.82–1.77	0.35
	(TT) (REF)			95∶311	1		
Mother alcohol use before pregnancy							
	GG		Yes	17∶23	3.72	1.63–8.50	**0.002**
	GT			58∶168	1.5	0.92–2.45	0.1
	TT	0.18^d^		58∶208	1		
	GG		No	8∶18	1.2	0.38–3.71	0.46
	GT			26∶92	0.78	0.40–1.51	0.76
	TT			37∶103	1		
Mother folic acid use before pregnancy							
	GG		Yes	12∶14	7.34	2.23–24.2	**0.001**
	GT			26∶72	2.39	1.06–5.40	0.04
	TT	**0.04** d		31∶121	1		
	GG		No	13∶27	1.46	0.63–3.37	0.38
	GT			57∶189	0.89	0.57–1.41	0.63
	TT			63∶183	1		
Father smoked during 2 years before pregnancy							
	GG		Yes	6∶11	1.35	0.42–4.36	0.61
	GT			23∶89	0.68	0.36–1.29	0.24
	TT	**0.05** d		40∶107	1		
	GG		No	18∶28	4.07	1.73–9.57	**0.001**
	GT			55∶151	1.84	1.08–3.15	0.03
	TT			47∶181	1		
Patient sex							
	GG		Male	13∶23	2.24	0.93–5.40	0.07
	GT			45∶137	1.2	0.71–2.02	0.49
	TT	0.90^4^		51∶167	1		
	GG		Female	12∶18	2.98	1.08–8.19	0.03
	GT			39∶123	1.21	0.67–2.16	0.53
	TT			44∶144	1		
**Patient age**				Mean Age (Cases:Controls)	OR	95% CI	P
	GG			5.2:5.7	0.88	0.72–1.07	0.2
	GT	0.31^4^		4.7:5.1	0.93	0.84–1.04	0.2
	TT			5.7:5.2	1		

a Case numbers reduced from GWAS according to the availability of both, parent genotypes and completed questionnaire; three matched controls per case were theoretically deduced from known parent genotypes.

b Referent group was genotype TT for all ORs.

c Likelihood ratio test p-value for main genetic effect (2df).

d likelihood ratio test p-value for additional interaction (2df) comparing environmental exposure categories.

Abbreviations: OR, Odds Ratio; CI, Confidence Interval; P, P-value.

An interaction was observed between the genotyped SNP at the *IKZF1* locus (rs4132601) and maternal use of folic acid pre-pregnancy (Table 2). There was a significantly increased risk of ALL among children with the homozygous minor genotype (GG) whose mothers used supplements; the OR was less elevated if they did not (*P* for interaction = 0.04).

We also observed interaction of rs4132601 genotype and father’s smoking prior to conception, whereby risk of ALL associated with the GG genotype was greater among children of non-smoking fathers (*P* for interaction = 0.05) (Table 2). In addition, there was some evidence that risk associated with the GG genotype at this SNP was elevated only if the mother drank alcohol before pregnancy (OR: 7.34 CI: 2.23–24.2); however, the interaction *P*-value was 0.18 (Table 2).

No interaction was evident for rs7089424 with parental exposure variables (Table S4 in File S1). No interaction was observed for variables sex and age with either SNP (*IKZF1* rs4132601 sex/age *P*s for interaction = 0.90/0.31, and *ARID5B* rs7089424 sex/age *P*s = 0.96/0.72) (Table 2 and Table S4 in File S1).

## Discussion

Our current study has demonstrated that variants in *ARID5B* and *IKZF1* are associated with childhood ALL in an Australian Caucasian cohort. This is consistent with former GWAS publications in similar cohorts [1]–[4]. Also in agreement with these studies is our finding that the strength of association was increased when analysis was confined to B-cell ALL versus controls. Our study was not large enough to reveal any other associations such as those previously reported at *CEBPE, CDKN2A, GATA3* and *BMI1-PIP4K2A* - loci which have not consistently been identified across multiple GWAS but which meta-analyses have validated [5], [21], [22].

The frequencies of the *ZNF704* SNPs in our case population were lower than that reported in the 1000 GENOMES phase 1 database but they were not replicated in the French data. This result could be due to population differences thereby suggesting a real effect; however, considering our cases were from an Anglo-Celtic background, and these SNPs were not identified in other such populations [1], it remains to be categorically defined as a risk variant.

The ARID5B transcription factor is important in embryogenesis and B-cell development [23] and *ARID5B* deletion mutations occur in leukemic cells. None of the significantly associated SNPs we identified at this locus are expression quantitative trait loci (eQTLs) according to Pritchard Lab and Blood eQTL browsers [16], [17], nor did they overlap enhancer binding sites annotated by ENCODE regulatory segmentation. Despite the four years since publication of the first childhood ALL GWAS, a dearth of information persists for the mechanisms responsible for *ARID5B* variants predisposing to childhood ALL.

The Ikaros transcription factor is restricted to the hemo-lymphopoietic system and is a key regulator of lymphocyte differentiation via chromatin remodelling. Our strongest associated SNP rs1110701, and other *IKZF1* SNPs identified in this study (rs10272724 and rs17133807), annotate to enhancer binding sites in the GM12878 B-Lymphocyte cell line according to ENCODE regulatory segmentation. These SNPs were identified as eQTLs acting in cis [16], [17] consistent with Papaemmanuil’s *et al.* [1] demonstrated attenuation of *IKZF1* gene expression with variant allele dosage at rs4132601 (which is in linkage disequilibrium with rs1110701 r^2^ = 0.802 D’ = 1; *P* in the current study 8.51×10^−8^). Interestingly, Meyer’s *et al*. [24] recent sequence analysis of *IKZF1* deletion breakpoints in leukemic cells revealed four recombination hotspots, one of which was observed in 20% of participants and localised to the untranslated region of exon 8, ∼1300 bp downstream from rs1110701. Thus, single variants or a combination of variants in this region may contribute to differential expression, splicing, or propensity for rearrangement in leukemic transformation. The full extent of these effects remains to be revealed.

Previous epidemiological studies have provided links between childhood ALL risk and environmental exposures to parents before and after conception. Additionally, certain genetic features of ALL are predominant in particular age groups, such as MLL rearrangements [25]. In the current study, we investigated the potential for interaction of parental exposures and child’s age and sex with child’s genotype. The most significant SNPs that were genotyped, rather than imputed, at *ARID5B* (rs7089424) and *IKZF1* (rs4132601) loci were used for the interaction analysis. This was to facilitate genotyping validation of the Illumina array technology and the extension of the study in a single step. Paternal smoking, maternal folate and alcohol use (each before conception) were investigated; however no interaction was apparent for *ARID5B* variant rs7089424 with any of these exposures. There were no interactions observed for the two variants with child’s sex and age, validating the findings of Linabery et al. [12].

A meta-analysis of maternal folic acid supplementation and risk of childhood ALL conducted by our affiliated Aus-ALL consortium, verified a protective effect for supplementation [10]. When we assessed interaction of maternal folic acid supplementation with *IKZF1* SNP rs4132601 (the most significant genotyped SNP at the locus), we observed elevated odds ratios for children with the risk (GG) genotype whose mother took supplements. This observation is contrary to expectations, and biologically plausible explanations for this direction of effect are lacking. Nonetheless, potential interaction between folate levels and genotype at *IKZF1* warrants further investigation since: i) transcription of the reduced folate carrier (*SLC19A1*) can be modulated by the balance of Ikaros activating and dominant-negative isoforms [26] and ii) increased circulating un-metabolised folic acid has demonstrated association with reduced natural killer cell cytotoxicity [27] which is in turn associated with cancer [28]. Additionally, our measure of folic acid exposure (supplementation-Yes/No) is without knowledge of possible dietary or metabolic folate sufficiency which could have influenced the observations. Implications of this finding suggest that folate quantification may be valuable prior to initiating supplementation.

We observed a similarly unexpected interaction whereby risk of childhood ALL was greater for children with rs4132601 risk (GG) genotype and non-smoking fathers (Table 2) despite a previous Aus-ALL epidemiological study [9] demonstrating that paternal smoking was a risk factor for childhood ALL. An interesting trend was observed whereby risk associated with *IKZF1* was elevated only for the GG genotype if the mother drank alcohol before pregnancy. While this may be a chance finding, it corroborates health recommendations to avoid alcohol during pregnancy.

Given the limited numbers of cases homozygous for the minor alleles for all interactions, it is possible that our findings for SNP rs4132601 occurred due to chance, nonetheless investigation in a larger population is required to elucidate whether any true interactions exist.

### Conclusions

We have replicated associations of *IKZF1* and *ARID5B* variants with childhood ALL in an Australian Caucasian population. The *IKZF1* variants identified were eQTLs and impacted enhancer sequences however, such was not the case for *ARID5B* variants. Furthermore, there was some evidence for gene-environment interaction for an *IKZF1* variant, with an apparently stronger genetic effect if the mother took folic acid or drank alcohol, or if the father did not smoke prior to pregnancy, but these may be explained by chance. These findings warrant further investigation in larger samples and may indicate that folate quantification may be valuable prior to initiating supplementation.

## Supporting Information

File S1 **Contains Methods S1, Tables S1–S4, Figures S1–S4.**
(DOCX)
