# Supplementary material for: Confirmation of Childhood Acute Lymphoblastic Leukemia Variants, ARID5B and IKZF1, and Interaction with Parental Environmental Exposures
Source: PLoS One. 2014 Oct 13;9(10):e110255. doi: 10.1371/journal.pone.0110255 (PMC4195717; doi:10.1371/journal.pone.0110255)
Supplement: File S1 — Contains Methods S1, Tables S1–S4, Figures S1–S4. (DOCX) [file pone.0110255.s001.docx]

**Supplementary Methods**

The starting population of cases, ages at diagnosis ranged from 0-15 years (mean = 6.1 years) and 244 were male and 197 were female. The mean age of Hunter Community Study Controls was 67 years [[McEvoy et al., 2010](#_ENREF_1_1)].

PLINK Software [[Purcell et al., 2007](#_ENREF_1_3)] was used for QC: Sample QC was applied excluding samples with discrepant gender, outlying heterozygosity or <95% call rate; SNP QC was applied first for each array version and again when merged, removing SNPs with >5% missingness, minor allele frequency <1% and HWE p-value<0.00001. Identity by descent (IBD) was assessed removing one individual of each pair with estimated IBD>0.1875. SNP missingness stringency was increased, removing SNPs with >2% missingness.

SNPs with differential missingness between cases/controls, and between arrays were excluded (P<1e-5), as were SNPs with significantly different allele frequencies between cases processed on different arrays (P<1e-8). Ancestry was inferred with a pruned set of 75867 SNPs using smartpca available in the EIGENSTRAT suite of programs [[Price et al., 2006](#_ENREF_1_2)] resulting in the exclusion of a further 57 individuals located more than six standard deviations from the mean ancestry estimate along the first ten principal components. SNPs positions were updated from build 36 to 37 and 1505 individuals (358 cases, 1192 controls) and 309117 SNPs were carried forward to imputation. Supplementary Table 1 details the composition of cases and controls following QC.

**Supplementary references**

Mcevoy M, Smith W, D'este C, Duke J, Peel R, Schofield P, Scott R, Byles J, Henry D, Ewald B, Hancock S, Smith D & Attia J 2010. Cohort profile: The Hunter Community Study. Int J Epidemiol 39**,** 1452-63.

Price AL, Patterson NJ, Plenge RM, Weinblatt ME, Shadick NA & Reich D 2006. Principal components analysis corrects for stratification in genome-wide association studies. Nat Genet 38**,** 904-9.

Purcell S, Neale B, Todd-Brown K, Thomas L, Ferreira MA, Bender D, Maller J, Sklar P, De Bakker PI, Daly MJ & Sham PC 2007. PLINK: a tool set for whole-genome association and population-based linkage analyses. Am J Hum Genet 81**,** 559-75.

**Supplementary Table S1: Study population demographics for post-QC GWAS population.**

| Characteristic | HCS Controls (n=1192) | ALL (n=358) |
| --- | --- | --- |
| Gender: count (% male) | 604 (50.7) | 195 (54.5) |
| Mean Age: years | 66.3 | 5.7 |
| ALL subtype: counts (%) |  |  |
| B-ALL | NA | 319 (89.1) |
| T-ALL | NA | 32 (8.9) |
| Other | NA | 7 (2) |


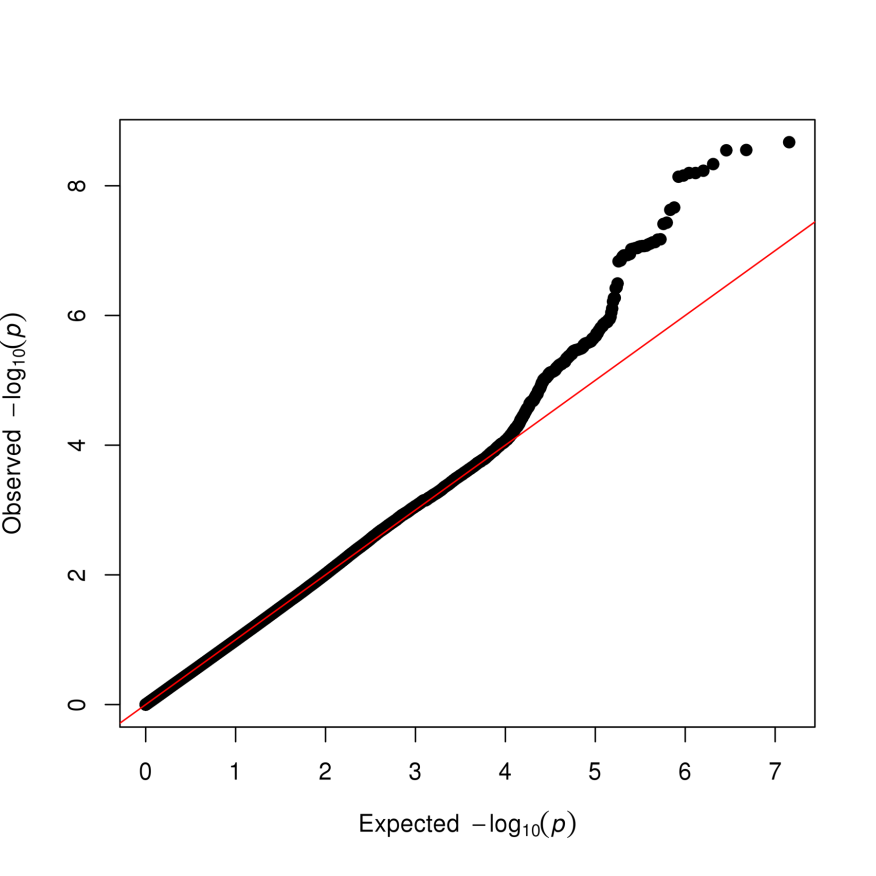


**Supplementary Figure S1:** Quantile-quantile plot comparing –log_10_-transformed *P*-values for GWAS of 358 ALL cases versus 1192 healthy controls using 7 162 141 SNPs (λ= 1.0006).


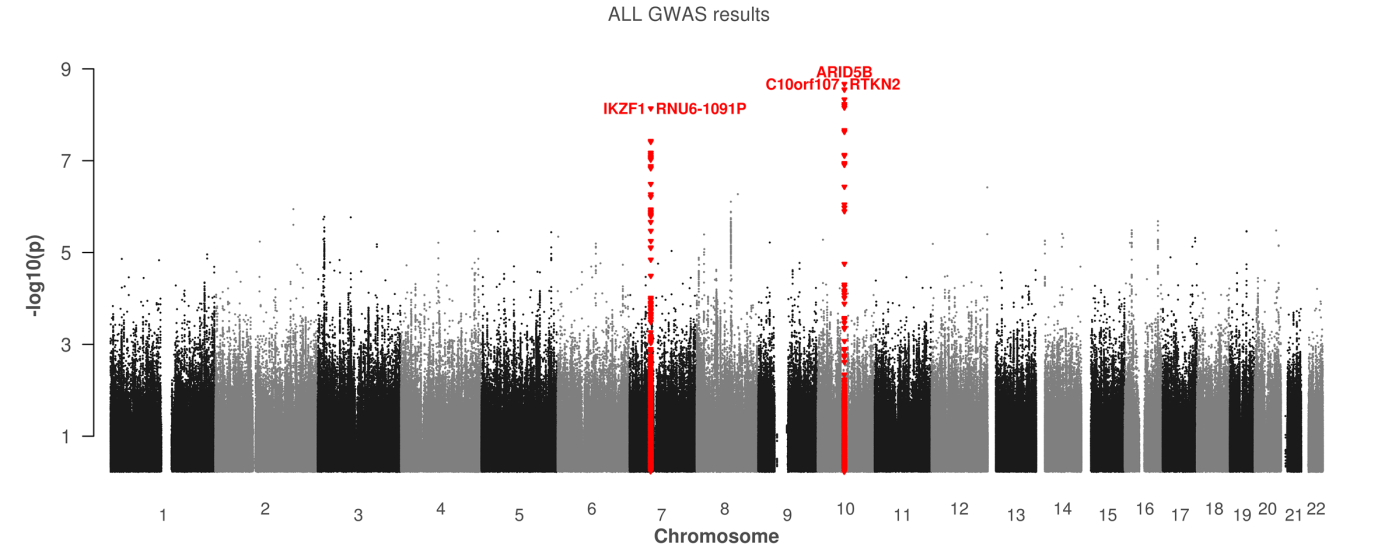


**Supplementary Figure S2:** Genome-wide association results for ALL (*P*=5×10^-8^). The plot shows –log_10_-transformed *P*-values for genotyped and imputed SNPs with respect to their physical genomic position.


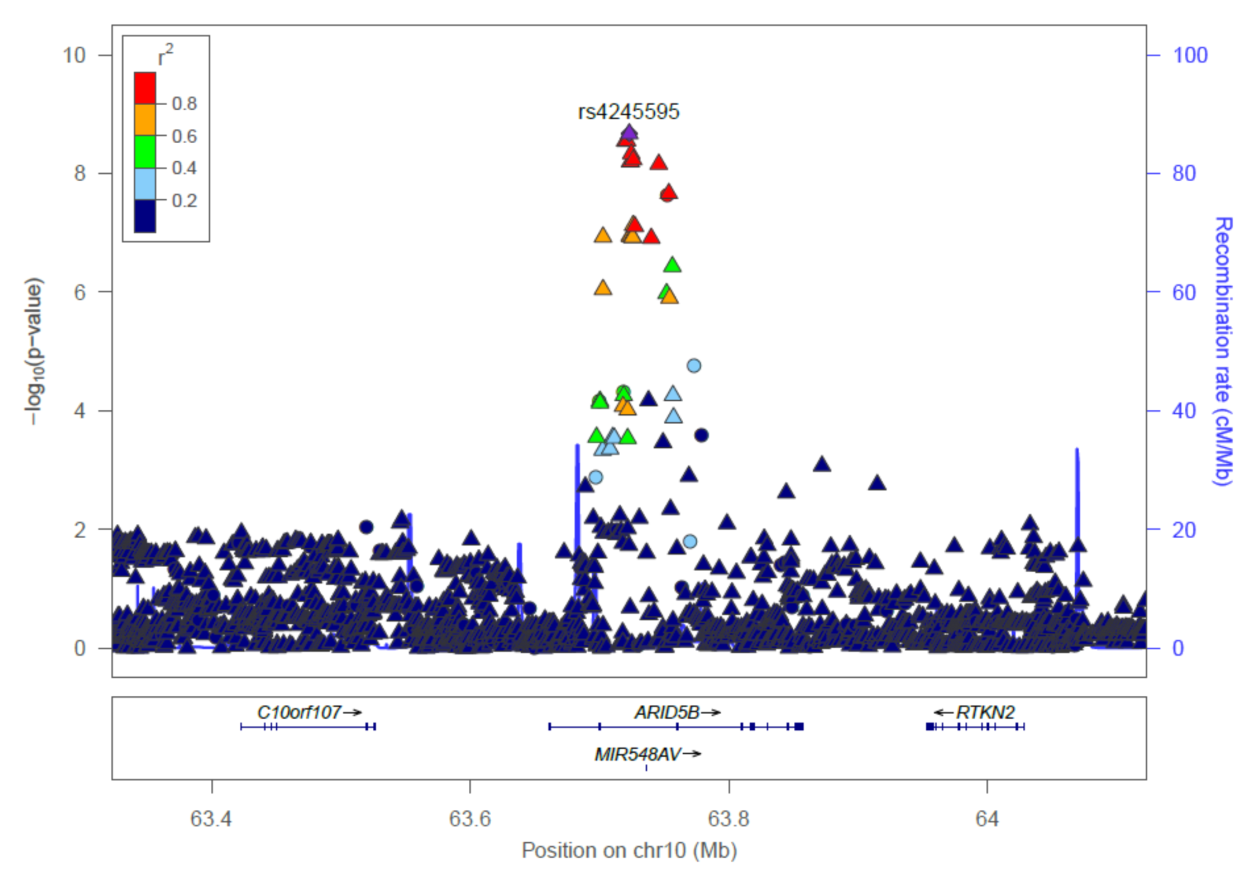

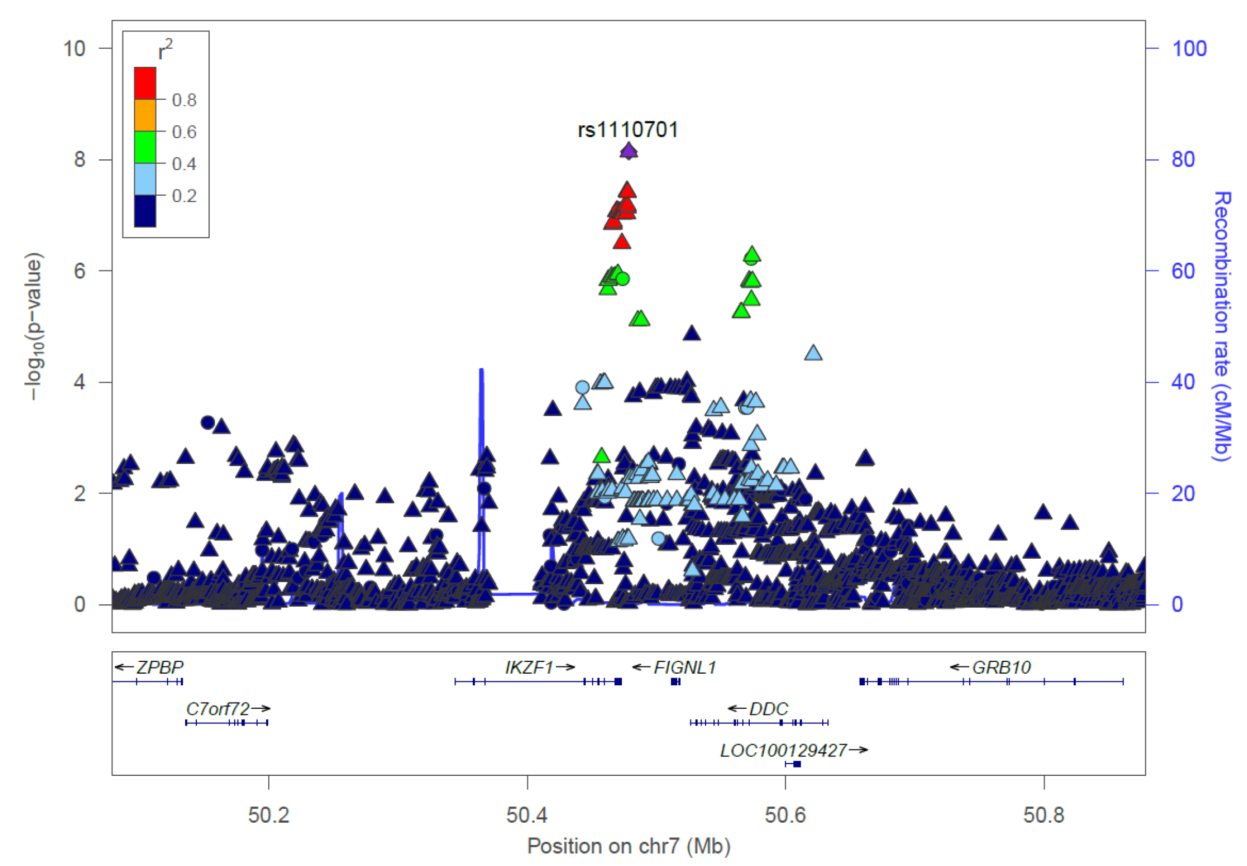


**Supplementary Figure S3: Regional association plots of *ARID5B* gene on chromosome 10 (top), *IKZF1*/*DDC* locus on chromosome 7 (bottom).** The most significant SNP in the respective region is plotted in purple and all other SNPs are coloured by their degree of linkage disequilibrium (r2) with the most significant SNP. Imputed SNPs are triangles and genotyped SNPs are circles. Local recombination rate estimates for the 1000 Genomes EUR samples are plotted against the secondary y-axis (blue line).


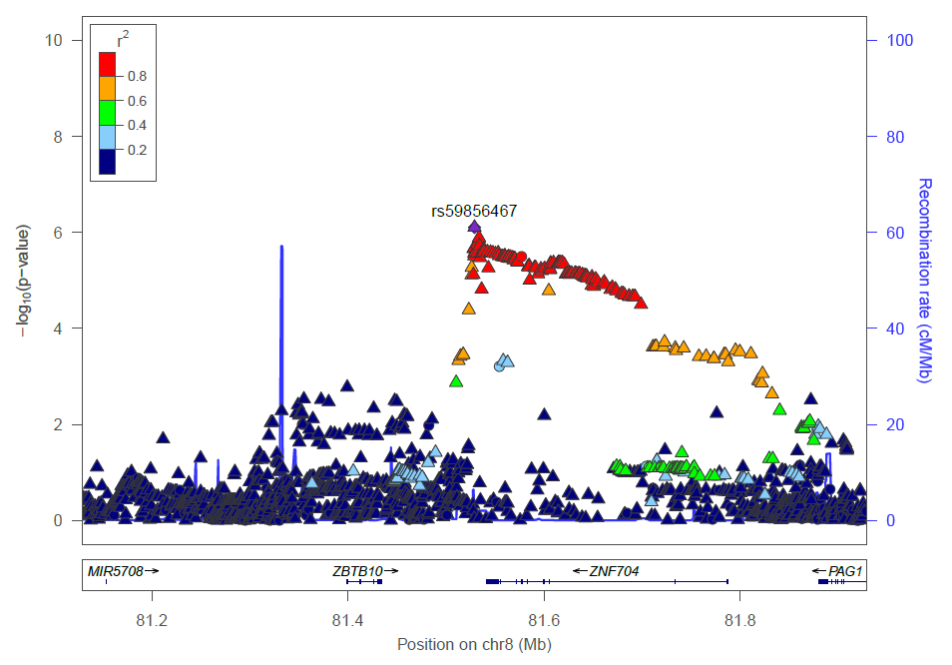


**Supplementary Figure S4: Regional association of chromosome 8 SNPs.** The most significant SNP is plotted in purple and all other SNPs are coloured by their degree of linkage disequilibrium (r2) with the most significant SNP. Imputed SNPs are triangles and genotyped SNPs are circles. Local recombination rate estimates for the 1000 Genomes EUR samples are plotted against the secondary y-axis (blue line).

**Supplementary Table S2: SNPs with 1x10^-5^< P < 5x10^-8^ from GWAS of Childhood Acute Lymphoblastic Leukemia.**

|  |  |  |  | |  | |  | | MAF | |  |  |  |  |  |  |  |
| --- | --- | --- | --- | --- | --- | --- | --- | --- | --- | --- | --- | --- | --- | --- | --- | --- | --- |
| Typed ^a^ | rs ID | Chr | Position | Gene (location) | | Proximal genes | | Alleles (A/B) ^b^ | Cases | Controls | Allelic OR (95% CI) ^c^ | P |  |  |  |  |  |
| --- | rs28696237 | 7 | 50475831 |  | | IKZF1,DDC,FIGNL1 | | C/G | 0.37 | 0.26 | 1.66 (1.39-1.98) | 6.69E-08 |  |  |  |  |  |
| --- | rs62445869 | 7 | 50477611 |  | | IKZF1,DDC,FIGNL1 | | G/A | 0.37 | 0.27 | 1.65 (1.38-1.96) | 6.78E-08 |  |  |  |  |  |
| --- | rs17133805 | 7 | 50477514 |  | | IKZF1,DDC,FIGNL1 | | T/G | 0.37 | 0.26 | 1.64 (1.38-1.96) | 7.38E-08 |  |  |  |  |  |
| --- | rs4245596 | 10 | 63725862 | ARID5B (intronic) | |  | | G/A | 0.54 | 0.43 | 0.65 (0.55-0.77) | 7.48E-08 |  |  |  |  |  |
| --- | rs4506592 | 10 | 63727187 | ARID5B (intronic) | |  | | A/G | 0.48 | 0.37 | 0.65 (0.55-0.77) | 7.78E-08 |  |  |  |  |  |
| --- | rs10264390 | 7 | 50475321 |  | | IKZF1,DDC,FIGNL1 | | T/C | 0.37 | 0.26 | 1.66 (1.39-1.98) | 8.04E-08 |  |  |  |  |  |
| --- | rs11552047 | 7 | 50469551 |  | | IKZF1,DDC,FIGNL1 | | C/T | 0.37 | 0.26 | 1.66 (1.39-1.98) | 8.40E-08 |  |  |  |  |  |
| rs4132601 | rs4132601 | 7 | 50470604 |  | | IKZF1,DDC,FIGNL1 | | T/G | 0.37 | 0.26 | 1.66 (1.39-1.98) | 8.51E-08 |  |  |  |  |  |
| --- | rs11980379 | 7 | 50469981 |  | | IKZF1,DDC,FIGNL1 | | T/C | 0.37 | 0.26 | 1.66 (1.39-1.98) | 8.52E-08 |  |  |  |  |  |
| --- | rs10278451 | 7 | 50468952 |  | | IKZF1,DDC,FIGNL1 | | G/T | 0.37 | 0.26 | 1.66 (1.39-1.98) | 8.59E-08 |  |  |  |  |  |
| --- | rs11980407 | 7 | 50471613 |  | | IKZF1,DDC,FIGNL1 | | G/A | 0.37 | 0.26 | 1.66 (1.39-1.98) | 8.74E-08 |  |  |  |  |  |
| --- | rs6964969 | 7 | 50473251 |  | | IKZF1,DDC,FIGNL1 | | A/G | 0.37 | 0.26 | 1.65 (1.39-1.97) | 9.13E-08 |  |  |  |  |  |
| --- | rs62445866 | 7 | 50472324 |  | | IKZF1,DDC,FIGNL1 | | G/A | 0.37 | 0.26 | 1.66 (1.39-1.98) | 9.13E-08 |  |  |  |  |  |
| --- | rs58923657 | 7 | 50472842 |  | | IKZF1,DDC,FIGNL1 | | C/T | 0.37 | 0.26 | 1.65 (1.39-1.97) | 9.29E-08 |  |  |  |  |  |
| --- | rs10230978 | 7 | 50477144 |  | | IKZF1,DDC,FIGNL1 | | G/A | 0.36 | 0.26 | 1.64 (1.37-1.96) | 9.39E-08 |  |  |  |  |  |
| --- | rs28462675 | 7 | 50473870 |  | | IKZF1,DDC,FIGNL1 | | A/G | 0.37 | 0.26 | 1.65 (1.38-1.97) | 9.51E-08 |  |  |  |  |  |
| --- | rs7908445 | 10 | 63723336 | ARID5B (intronic) | |  | | T/C | 0.53 | 0.43 | 0.66 (0.56-0.78) | 1.13E-07 |  |  |  |  |  |
| --- | rs7923074 | 10 | 63723440 | ARID5B (intronic) | |  | | A/C | 0.53 | 0.43 | 0.66 (0.56-0.78) | 1.14E-07 |  |  |  |  |  |
| --- | rs10740053 | 10 | 63702584 | ARID5B (intronic) | |  | | C/T | 0.44 | 0.34 | 0.65 (0.54-0.76) | 1.17E-07 |  |  |  |  |  |
| --- | rs10821938 | 10 | 63724773 | ARID5B (intronic) | |  | | A/C | 0.53 | 0.43 | 0.66 (0.56-0.78) | 1.17E-07 |  |  |  |  |  |
| --- | rs4948494 | 10 | 63725424 | ARID5B (intronic) | |  | | G/C | 0.53 | 0.43 | 0.66 (0.56-0.78) | 1.19E-07 |  |  |  |  |  |
| --- | rs4948493 | 10 | 63725372 | ARID5B (intronic) | |  | | A/G | 0.53 | 0.43 | 0.66 (0.56-0.78) | 1.19E-07 |  |  |  |  |  |
| --- | rs10761598 | 10 | 63739860 | ARID5B (intronic) | |  | | T/A | 0.48 | 0.37 | 0.65 (0.55-0.77) | 1.23E-07 |  |  |  |  |  |
| --- | rs6960400 | 7 | 50466797 |  | | IKZF1,DDC,FIGNL1 | | A/G | 0.37 | 0.27 | 1.64 (1.37-1.95) | 1.31E-07 |  |  |  |  |  |
| --- | rs11978267 | 7 | 50466304 |  | | IKZF1,DDC,FIGNL1 | | A/G | 0.37 | 0.26 | 1.63 (1.37-1.95) | 1.42E-07 |  |  |  |  |  |
| --- | rs6973210 | 7 | 50466695 |  | | IKZF1,DDC,FIGNL1 | | G/A | 0.37 | 0.27 | 1.63 (1.37-1.95) | 1.43E-07 |  |  |  |  |  |
| --- | rs62447205 | 7 | 50465830 |  | | IKZF1,DDC,FIGNL1 | | A/G | 0.37 | 0.27 | 1.63 (1.37-1.95) | 1.46E-07 |  |  |  |  |  |
| --- | rs6956014 | 7 | 50473290 |  | | IKZF1,DDC,FIGNL1 | | T/C | 0.36 | 0.26 | 1.62 (1.36-1.94) | 3.21E-07 |  |  |  |  |  |
| --- | rs2893877 | 10 | 63756199 | ARID5B (intronic) | |  | | A/G | 0.57 | 0.46 | 1.55 (1.31-1.84) | 3.69E-07 |  |  |  |  |  |
| --- | rs80128697 | 12 | 132661466 |  | | DDX51,EP400NL,GALNT9,EP400,NOC4L | | G/A | 0.09 | 0.05 | 2.06 (1.5-2.84) | 3.81E-07 |  |  |  |  |  |
| --- | rs184472327 | 8 | 98135357 |  | | CPQ | | A/G | 0.06 | 0.04 | 1.79 (1.23-2.59) | 5.36E-07 |  |  |  |  |  |
| --- | rs1817074 | 7 | 50574012 | DDC (intronic) | |  | | T/C | 0.45 | 0.35 | 1.51 (1.28-1.79) | 5.39E-07 |  |  |  |  |  |
| rs7809758 | rs7809758 | 7 | 50573333 | DDC (intronic) | |  | | A/G | 0.45 | 0.35 | 1.51 (1.28-1.79) | 6.10E-07 |  |  |  |  |  |
| --- | rs59856467 | 8 | 81528928 |  | | SLC25A51P3,OCIAD2P1,CKS1BP7,RPSAP47,ZNF704,ZBTB10 | | A/G | 0.03 | 0.07 | 0.34 (0.21-0.55) | 7.82E-07 |  |  |  |  |  |
| --- | rs10740054 | 10 | 63702586 | ARID5B (intronic) | |  | | T/A | 0.48 | 0.38 | 0.68 (0.57-0.8) | 9.00E-07 |  |  |  |  |  |
| --- | rs10821939 | 10 | 63751748 | ARID5B (intronic) | |  | | G/A | 0.54 | 0.44 | 0.68 (0.57-0.8) | 1.05E-06 |  |  |  |  |  |
| --- | rs114566971 | 2 | 185614749 | ZNF804A (intronic) | |  | | A/T | 0.05 | 0.02 | 2.39 (1.55-3.68) | 1.13E-06 |  |  |  |  |  |
| --- | rs62447208 | 7 | 50470156 |  | | IKZF1,DDC,FIGNL1 | | G/C | 0.27 | 0.18 | 1.7 (1.4-2.07) | 1.15E-06 |  |  |  |  |  |
| --- | rs62447207 | 7 | 50470138 |  | | IKZF1,DDC,FIGNL1 | | G/T | 0.27 | 0.18 | 1.7 (1.39-2.06) | 1.18E-06 |  |  |  |  |  |
| --- | rs9414758 | 10 | 63754024 | ARID5B (intronic) | |  | | T/A | 0.47 | 0.37 | 1.49 (1.26-1.76) | 1.26E-06 |  |  |  |  |  |
| --- | rs55981617 | 7 | 50465206 |  | | IKZF1,DDC,FIGNL1 | | G/A | 0.26 | 0.18 | 1.68 (1.38-2.05) | 1.28E-06 |  |  |  |  |  |
| --- | rs61731355 | 7 | 50467767 |  | | IKZF1,DDC,FIGNL1 | | C/A | 0.27 | 0.18 | 1.69 (1.38-2.05) | 1.28E-06 |  |  |  |  |  |
| --- | rs6473237 | 8 | 81533591 |  | | SLC25A51P3,OCIAD2P1,CKS1BP7,RPSAP47,ZNF704,ZBTB10 | | T/A | 0.03 | 0.08 | 0.37 (0.23-0.58) | 1.29E-06 |  |  |  |  |  |
| --- | rs6473236 | 8 | 81533311 |  | | SLC25A51P3,OCIAD2P1,CKS1BP7,RPSAP47,ZNF704,ZBTB10 | | C/T | 0.03 | 0.07 | 0.35 (0.22-0.57) | 1.34E-06 |  |  |  |  |  |
| --- | rs56156635 | 7 | 50466276 |  | | IKZF1,DDC,FIGNL1 | | G/A | 0.26 | 0.18 | 1.68 (1.38-2.05) | 1.35E-06 |  |  |  |  |  |
| rs6944602 | rs6944602 | 7 | 50473751 |  | | IKZF1,DDC,FIGNL1 | | G/A | 0.27 | 0.18 | 1.69 (1.39-2.06) | 1.37E-06 |  |  |  |  |  |
| --- | rs6952409 | 7 | 50462935 |  | | IKZF1,DDC,FIGNL1 | | G/A | 0.26 | 0.18 | 1.68 (1.38-2.04) | 1.47E-06 |  |  |  |  |  |
| --- | rs10215297 | 7 | 50462498 |  | | IKZF1,DDC,FIGNL1 | | A/T | 0.26 | 0.18 | 1.67 (1.38-2.04) | 1.49E-06 |  |  |  |  |  |
| --- | rs11575375 | 7 | 50571779 | DDC (intronic) | |  | | G/A | 0.44 | 0.34 | 1.49 (1.25-1.76) | 1.52E-06 |  |  |  |  |  |
| --- | rs2198144 | 7 | 50574254 | DDC (intronic) | |  | | A/C | 0.44 | 0.35 | 1.49 (1.26-1.77) | 1.55E-06 |  |  |  |  |  |
| --- | rs4947584 | 7 | 50572709 | DDC (intronic) | |  | | A/T | 0.44 | 0.34 | 1.49 (1.25-1.76) | 1.61E-06 |  |  |  |  |  |
| --- | rs4685272 | 3 | 15951444 |  | | LOC101927674,LOC101927725,IMPDH1P8,ANKRD28,MIR563 | | C/A | 0.28 | 0.21 | 0.67 (0.55-0.81) | 1.67E-06 |  |  |  |  |  |
| --- | rs62260387 | 3 | 78678920 | ROBO1 (intronic) | |  | | A/G | 0.05 | 0.03 | 2.12 (1.4-3.2) | 1.71E-06 |  |  |  |  |  |
| --- | rs11989711 | 8 | 81531810 |  | | SLC25A51P3,OCIAD2P1,CKS1BP7,RPSAP47,ZNF704,ZBTB10 | | T/C | 0.03 | 0.07 | 0.35 (0.22-0.57) | 1.78E-06 |  |  |  |  |  |
| --- | rs113979561 | 8 | 81533835 |  | | SLC25A51P3,OCIAD2P1,CKS1BP7,RPSAP47,ZNF704,ZBTB10 | | T/C | 0.03 | 0.07 | 0.35 (0.22-0.57) | 1.88E-06 |  |  |  |  |  |
| --- | rs3773267 | 3 | 13662397 | FBLN2 (intronic) | |  | | T/C | 0.05 | 0.02 | 2.63 (1.71-4.04) | 1.89E-06 |  |  |  |  |  |
| --- | rs73691949 | 8 | 81534025 |  | | SLC25A51P3,OCIAD2P1,CKS1BP7,RPSAP47,ZNF704,ZBTB10 | | G/T | 0.03 | 0.07 | 0.35 (0.22-0.57) | 1.90E-06 |  |  |  |  |  |
| --- | rs55660615 | 16 | 79094542 | WWOX (intronic) | |  | | C/G | 0.05 | 0.02 | 2.37 (1.52-3.7) | 2.07E-06 |  |  |  |  |  |
| --- | rs28545210 | 16 | 79097591 | WWOX (intronic) | |  | | C/G | 0.05 | 0.02 | 2.37 (1.52-3.71) | 2.09E-06 |  |  |  |  |  |
| --- | rs16907900 | 8 | 81528234 |  | | SLC25A51P3,OCIAD2P1,CKS1BP7,RPSAP47,ZNF704,ZBTB10 | | A/C | 0.03 | 0.07 | 0.36 (0.22-0.58) | 2.13E-06 |  |  |  |  |  |
| --- | rs10216316 | 7 | 50462418 |  | | IKZF1,DDC,FIGNL1 | | C/T | 0.27 | 0.18 | 1.66 (1.37-2.03) | 2.16E-06 |  |  |  |  |  |
| --- | rs16907894 | 8 | 81528112 |  | | SLC25A51P3,OCIAD2P1,CKS1BP7,RPSAP47,ZNF704,ZBTB10 | | T/C | 0.03 | 0.07 | 0.36 (0.22-0.58) | 2.19E-06 |  |  |  |  |  |
| --- | rs111565089 | 8 | 81538469 |  | | SLC25A51P3,OCIAD2P1,CKS1BP7,RPSAP47,ZNF704,ZBTB10 | | T/C | 0.03 | 0.07 | 0.35 (0.22-0.57) | 2.29E-06 |  |  |  |  |  |
| --- | rs113266056 | 8 | 81538471 |  | | SLC25A51P3,OCIAD2P1,CKS1BP7,RPSAP47,ZNF704,ZBTB10 | | A/G | 0.03 | 0.07 | 0.35 (0.22-0.57) | 2.29E-06 |  |  |  |  |  |
| --- | rs111559015 | 8 | 81538474 |  | | SLC25A51P3,OCIAD2P1,CKS1BP7,RPSAP47,ZNF704,ZBTB10 | | A/C | 0.03 | 0.07 | 0.35 (0.22-0.57) | 2.29E-06 |  |  |  |  |  |
| --- | rs7014034 | 8 | 81537606 |  | | SLC25A51P3,OCIAD2P1,CKS1BP7,RPSAP47,ZNF704,ZBTB10 | | C/T | 0.03 | 0.07 | 0.35 (0.22-0.57) | 2.38E-06 |  |  |  |  |  |
| --- | rs79895553 | 2 | 185593635 | ZNF804A (intronic) | |  | | G/A | 0.05 | 0.02 | 2.27 (1.49-3.46) | 2.49E-06 |  |  |  |  |  |
| --- | rs73260945 | 8 | 81542089 | ZNF704 (utr-variant-3-prime) | |  | | C/T | 0.03 | 0.07 | 0.35 (0.22-0.57) | 2.53E-06 |  |  |  |  |  |
| --- | rs112272342 | 8 | 81538478 |  | | SLC25A51P3,OCIAD2P1,CKS1BP7,RPSAP47,ZNF704,ZBTB10 | | G/T | 0.03 | 0.07 | 0.35 (0.22-0.57) | 2.53E-06 |  |  |  |  |  |
| --- | rs28697115 | 16 | 79100288 | WWOX (intronic) | |  | | A/G | 0.05 | 0.02 | 2.37 (1.51-3.71) | 2.56E-06 |  |  |  |  |  |
| --- | rs78171982 | 8 | 81529387 |  | | SLC25A51P3,OCIAD2P1,CKS1BP7,RPSAP47,ZNF704,ZBTB10 | | A/T | 0.03 | 0.07 | 0.36 (0.22-0.58) | 2.56E-06 |  |  |  |  |  |
| --- | rs17473194 | 8 | 81545264 | ZNF704 (utr-variant-3-prime) | |  | | G/A | 0.03 | 0.07 | 0.35 (0.22-0.57) | 2.59E-06 |  |  |  |  |  |
| rs7000234 | rs7000234 | 8 | 81537114 |  | | SLC25A51P3,OCIAD2P1,CKS1BP7,RPSAP47,ZNF704,ZBTB10 | | G/A | 0.03 | 0.07 | 0.35 (0.22-0.57) | 2.62E-06 |  |  |  |  |  |
| --- | rs55967237 | 8 | 81536307 |  | | SLC25A51P3,OCIAD2P1,CKS1BP7,RPSAP47,ZNF704,ZBTB10 | | C/T | 0.03 | 0.07 | 0.35 (0.22-0.57) | 2.63E-06 |  |  |  |  |  |
| --- | rs80087908 | 8 | 81531692 |  | | SLC25A51P3,OCIAD2P1,CKS1BP7,RPSAP47,ZNF704,ZBTB10 | | T/A | 0.03 | 0.07 | 0.36 (0.22-0.58) | 2.65E-06 |  |  |  |  |  |
| --- | rs10504721 | 8 | 81553122 | ZNF704 (utr-variant-3-prime) | |  | | G/A | 0.03 | 0.07 | 0.35 (0.22-0.57) | 2.67E-06 |  |  |  |  |  |
| --- | rs77184226 | 8 | 81547746 | ZNF704 (utr-variant-3-prime) | |  | | C/T | 0.03 | 0.07 | 0.35 (0.22-0.57) | 2.67E-06 |  |  |  |  |  |
| --- | rs73260930 | 8 | 81529452 |  | | SLC25A51P3,OCIAD2P1,CKS1BP7,RPSAP47,ZNF704,ZBTB10 | | G/A | 0.03 | 0.07 | 0.36 (0.22-0.58) | 2.68E-06 |  |  |  |  |  |
| --- | rs73260928 | 8 | 81528978 |  | | SLC25A51P3,OCIAD2P1,CKS1BP7,RPSAP47,ZNF704,ZBTB10 | | A/G | 0.03 | 0.07 | 0.36 (0.22-0.58) | 2.69E-06 |  |  |  |  |  |
| --- | rs73691950 | 8 | 81535574 |  | | SLC25A51P3,OCIAD2P1,CKS1BP7,RPSAP47,ZNF704,ZBTB10 | | T/C | 0.03 | 0.07 | 0.35 (0.22-0.57) | 2.71E-06 |  |  |  |  |  |
| --- | rs73260924 | 8 | 81528869 |  | | SLC25A51P3,OCIAD2P1,CKS1BP7,RPSAP47,ZNF704,ZBTB10 | | C/G | 0.03 | 0.07 | 0.36 (0.22-0.58) | 2.73E-06 |  |  |  |  |  |
| --- | rs75309399 | 8 | 81550269 | ZNF704 (utr-variant-3-prime) | |  | | A/G | 0.03 | 0.07 | 0.35 (0.22-0.57) | 2.78E-06 |  |  |  |  |  |
| --- | rs138392874 | 3 | 15952888 |  | | LOC101927674,LOC101927725,IMPDH1P8,ANKRD28,MIR563 | | G/T | 0.32 | 0.25 | 0.69 (0.58-0.83) | 2.85E-06 |  |  |  |  |  |
| --- | rs2166757 | 3 | 15954985 |  | | LOC101927674,LOC101927725,IMPDH1P8,ANKRD28,MIR563 | | A/G | 0.26 | 0.19 | 0.65 (0.54-0.8) | 2.85E-06 |  |  |  |  |  |
| --- | rs113410521 | 8 | 81553857 | ZNF704 (intronic) | |  | | C/T | 0.03 | 0.07 | 0.35 (0.22-0.57) | 3.05E-06 |  |  |  |  |  |
| --- | rs7840018 | 8 | 81559807 | ZNF704 (intronic) | |  | | A/T | 0.03 | 0.07 | 0.35 (0.22-0.57) | 3.06E-06 |  |  |  |  |  |
| --- | rs16907912 | 8 | 81528714 |  | | SLC25A51P3,OCIAD2P1,CKS1BP7,RPSAP47,ZNF704,ZBTB10 | | A/C | 0.03 | 0.07 | 0.36 (0.22-0.58) | 3.08E-06 |  |  |  |  |  |
| --- | rs148245574 | 8 | 81556936 | ZNF704 (intronic) | |  | | G/A | 0.03 | 0.07 | 0.35 (0.22-0.57) | 3.17E-06 |  |  |  |  |  |
| --- | rs112966494 | 8 | 81557796 | ZNF704 (intronic) | |  | | C/T | 0.03 | 0.07 | 0.35 (0.22-0.57) | 3.20E-06 |  |  |  |  |  |
| --- | rs181645483 | 8 | 81563754 | ZNF704 (intronic) | |  | | G/A | 0.03 | 0.07 | 0.35 (0.22-0.57) | 3.21E-06 |  |  |  |  |  |
| rs7018449 | rs7018449 | 8 | 81576965 | ZNF704 (intronic) | |  | | T/C | 0.03 | 0.07 | 0.36 (0.22-0.57) | 3.24E-06 |  |  |  |  |  |
| --- | rs79237313 | 8 | 81559246 | ZNF704 (intronic) | |  | | G/A | 0.03 | 0.07 | 0.35 (0.22-0.57) | 3.25E-06 |  |  |  |  |  |
| --- | rs34575794 | 16 | 16873816 |  | |  | | G/T | 0.29 | 0.23 | 1.42 (1.18-1.71) | 3.27E-06 |  |  |  |  |  |
| --- | rs78182567 | 8 | 81560209 | ZNF704 (intronic) | |  | | A/G | 0.03 | 0.07 | 0.35 (0.22-0.57) | 3.28E-06 |  |  |  |  |  |
| --- | rs9773390 | 8 | 81565692 | ZNF704 (intronic) | |  | | T/C | 0.03 | 0.07 | 0.35 (0.22-0.57) | 3.29E-06 |  |  |  |  |  |
| rs707544 | rs707544 | 20 | 51283095 |  | | TRNAI30P, | | C/T | 0.06 | 0.12 | 2.12 (1.52-2.94) | 3.31E-06 |  |  |  |  |  |
| --- | rs28522573 | 16 | 79086855 | WWOX (intronic) | |  | | G/T | 0.05 | 0.02 | 2.33 (1.49-3.63) | 3.33E-06 |  |  |  |  |  |
| --- | rs16907926 | 8 | 81534679 |  | | SLC25A51P3,OCIAD2P1,CKS1BP7,RPSAP47,ZNF704,ZBTB10 | | A/G | 0.03 | 0.07 | 0.36 (0.22-0.58) | 3.35E-06 |  |  |  |  |  |
| --- | rs58612109 | 7 | 50573455 | DDC (intronic) | |  | | G/A | 0.42 | 0.33 | 1.47 (1.24-1.75) | 3.37E-06 |  |  |  |  |  |
| --- | rs4684260 | 3 | 15796399 | ANKRD28 (intronic) | |  | | A/G | 0.32 | 0.24 | 0.67 (0.56-0.8) | 3.38E-06 |  |  |  |  |  |
| --- | rs115288238 | 8 | 81562509 | ZNF704 (intronic) | |  | | G/A | 0.03 | 0.07 | 0.35 (0.22-0.57) | 3.38E-06 |  |  |  |  |  |
| --- | rs148600161 | 8 | 81562533 | ZNF704 (intronic) | |  | | G/A | 0.03 | 0.07 | 0.35 (0.22-0.57) | 3.38E-06 |  |  |  |  |  |
| --- | rs150102979 | 8 | 81563308 | ZNF704 (intronic) | |  | | A/G | 0.03 | 0.07 | 0.35 (0.22-0.57) | 3.42E-06 |  |  |  |  |  |
| --- | rs73691962 | 8 | 81567807 | ZNF704 (intronic) | |  | | C/T | 0.03 | 0.07 | 0.35 (0.22-0.57) | 3.42E-06 |  |  |  |  |  |
| --- | rs72698193 | 4 | 174915374 |  | |  | | A/G | 0.12 | 0.08 | 1.64 (1.26-2.14) | 3.42E-06 |  |  |  |  |  |
| --- | rs73040091 | 19 | 40038686 |  | | TDGF1P7,RPS29P24,RPS29P25,RPS29P26,DLL3,EID2B,EID2,LGALS13,SUPT5H,TIMM50 | | G/C | 0.11 | 0.06 | 1.85 (1.39-2.46) | 3.43E-06 |  |  |  |  |  |
| --- | rs73691963 | 8 | 81568044 | ZNF704 (intronic) | |  | | C/T | 0.03 | 0.07 | 0.35 (0.22-0.57) | 3.44E-06 |  |  |  |  |  |
| --- | rs266918 | 5 | 39253573 | FYB (intronic) | |  | | T/A | 0.07 | 0.04 | 0.59 (0.42-0.83) | 3.46E-06 |  |  |  |  |  |
| --- | rs113594760 | 19 | 40017516 |  | | TDGF1P7,RPS29P24,RPS29P25,RPS29P26,DLL3,EID2B,EID2,LGALS13,RPS16,PLEKHG2,SUPT5H,TIMM50 | | C/G | 0.09 | 0.05 | 2.03 (1.47-2.8) | 3.49E-06 |  |  |  |  |  |
| --- | rs7638070 | 3 | 15953365 |  | | LOC101927674,LOC101927725,IMPDH1P8,ANKRD28,MIR563 | | G/A | 0.28 | 0.20 | 0.66 (0.55-0.8) | 3.51E-06 |  |  |  |  |  |
| --- | rs146167065 | 8 | 81565115 | ZNF704 (intronic) | |  | | G/T | 0.03 | 0.07 | 0.35 (0.22-0.57) | 3.51E-06 |  |  |  |  |  |
| --- | rs7018189 | 8 | 81569563 | ZNF704 (intronic) | |  | | T/C | 0.03 | 0.07 | 0.35 (0.22-0.57) | 3.53E-06 |  |  |  |  |  |
| --- | rs6998930 | 8 | 81570177 | ZNF704 (intronic) | |  | | A/G | 0.03 | 0.07 | 0.35 (0.22-0.57) | 3.55E-06 |  |  |  |  |  |
| --- | rs2961869 | 5 | 165917609 |  | |  | | C/G | 0.27 | 0.19 | 0.63 (0.52-0.77) | 3.61E-06 |  |  |  |  |  |
| --- | rs78588483 | 8 | 81568883 | ZNF704 (intronic) | |  | | T/C | 0.03 | 0.07 | 0.36 (0.22-0.58) | 3.72E-06 |  |  |  |  |  |
| --- | rs113945715 | 8 | 81568933 | ZNF704 (intronic) | |  | | T/C | 0.03 | 0.07 | 0.36 (0.22-0.58) | 3.72E-06 |  |  |  |  |  |
| --- | rs7194246 | 16 | 16874412 |  | |  | | G/A | 0.30 | 0.23 | 1.42 (1.18-1.71) | 3.73E-06 |  |  |  |  |  |
| --- | rs112426089 | 14 | 61463958 | SLC38A6 (intronic) | |  | | C/T | 0.08 | 0.04 | 1.99 (1.42-2.79) | 3.94E-06 |  |  |  |  |  |
| --- | rs78292237 | 14 | 61461779 | SLC38A6 (intronic) | |  | | T/C | 0.08 | 0.04 | 1.99 (1.42-2.79) | 3.94E-06 |  |  |  |  |  |
| --- | rs67713780 | 16 | 16871555 |  | |  | | T/G | 0.31 | 0.24 | 1.42 (1.18-1.71) | 3.97E-06 |  |  |  |  |  |
| --- | rs7487848 | 12 | 132665896 |  | | DDX51,EP400NL,GALNT9,NOC4L | | C/T | 0.07 | 0.03 | 2.24 (1.53-3.26) | 3.99E-06 |  |  |  |  |  |
| --- | rs67962801 | 16 | 16873871 |  | |  | | C/T | 0.29 | 0.23 | 1.41 (1.17-1.71) | 4.00E-06 |  |  |  |  |  |
| --- | rs117458235 | 8 | 17897643 |  | | MRPS18CP3,ASAH1,PCM1 | | C/A | 0.06 | 0.02 | 2.37 (1.57-3.59) | 4.05E-06 |  |  |  |  |  |
| --- | rs77185104 | 8 | 81572365 | ZNF704 (intronic) | |  | | T/C | 0.03 | 0.07 | 0.36 (0.22-0.58) | 4.07E-06 |  |  |  |  |  |
| --- | rs76774592 | 8 | 81572914 | ZNF704 (intronic) | |  | | A/G | 0.03 | 0.07 | 0.36 (0.22-0.58) | 4.17E-06 |  |  |  |  |  |
| --- | rs16907976 | 8 | 81614546 | ZNF704 (intronic) | |  | | T/C | 0.03 | 0.07 | 0.38 (0.24-0.6) | 4.22E-06 |  |  |  |  |  |
| --- | rs112833067 | 8 | 81615749 | ZNF704 (intronic) | |  | | A/G | 0.03 | 0.07 | 0.38 (0.24-0.6) | 4.24E-06 |  |  |  |  |  |
| --- | rs73273032 | 8 | 81616762 | ZNF704 (intronic) | |  | | A/G | 0.03 | 0.07 | 0.38 (0.24-0.6) | 4.25E-06 |  |  |  |  |  |
| --- | rs73273038 | 8 | 81618519 | ZNF704 (intronic) | |  | | A/G | 0.03 | 0.07 | 0.38 (0.24-0.6) | 4.27E-06 |  |  |  |  |  |
| --- | rs73273004 | 8 | 81609442 | ZNF704 (intronic) | |  | | T/C | 0.03 | 0.07 | 0.38 (0.24-0.6) | 4.28E-06 |  |  |  |  |  |
| --- | rs74997081 | 8 | 81606467 | ZNF704 (intronic) | |  | | T/C | 0.03 | 0.07 | 0.38 (0.24-0.6) | 4.35E-06 |  |  |  |  |  |
| --- | rs10504720 | 8 | 81606686 | ZNF704 (intronic) | |  | | G/A | 0.03 | 0.07 | 0.38 (0.24-0.6) | 4.48E-06 |  |  |  |  |  |
| --- | rs2318108 | 6 | 1783495 | GMDS (intronic) | |  | | A/G | 0.32 | 0.40 | 0.7 (0.58-0.83) | 4.52E-06 |  |  |  |  |  |
| --- | rs112633592 | 8 | 81613334 | ZNF704 (intronic) | |  | | T/C | 0.03 | 0.07 | 0.38 (0.24-0.6) | 4.52E-06 |  |  |  |  |  |
| --- | rs35163915 | 16 | 16870295 |  | |  | | T/G | 0.30 | 0.24 | 1.42 (1.18-1.71) | 4.53E-06 |  |  |  |  |  |
| --- | rs75106560 | 8 | 81614773 | ZNF704 (intronic) | |  | | A/G | 0.03 | 0.07 | 0.38 (0.24-0.6) | 4.55E-06 |  |  |  |  |  |
| --- | rs75269337 | 8 | 81609180 | ZNF704 (intronic) | |  | | C/T | 0.03 | 0.07 | 0.38 (0.24-0.61) | 4.58E-06 |  |  |  |  |  |
| --- | rs79431906 | 8 | 81620488 | ZNF704 (intronic) | |  | | C/T | 0.03 | 0.07 | 0.38 (0.24-0.61) | 4.64E-06 |  |  |  |  |  |
| --- | rs11850679 | 14 | 63933801 | PPP2R5E (intronic) | |  | | G/A | 0.09 | 0.05 | 1.78 (1.31-2.44) | 4.79E-06 |  |  |  |  |  |
| --- | rs56919062 | 17 | 77258897 | RBFOX3 (intronic) | |  | | G/C | 0.25 | 0.19 | 1.38 (1.14-1.69) | 4.82E-06 |  |  |  |  |  |
| --- | rs7841261 | 8 | 81584548 | ZNF704 (intronic) | |  | | G/C | 0.03 | 0.07 | 0.36 (0.22-0.58) | 4.89E-06 |  |  |  |  |  |
| --- | rs6442555 | 3 | 15937416 |  | | LOC101927674,LOC101927725,IMPDH1P8,ANKRD28,MIR563 | | T/C | 0.28 | 0.21 | 0.67 (0.55-0.81) | 4.93E-06 |  |  |  |  |  |
| --- | rs7630380 | 3 | 13663149 | FBLN2 (intronic) | |  | | C/A | 0.05 | 0.02 | 2.6 (1.67-4.04) | 5.18E-06 |  |  |  |  |  |
| --- | rs112232110 | 8 | 81583418 | ZNF704 (intronic) | |  | | A/C | 0.03 | 0.07 | 0.36 (0.22-0.58) | 5.20E-06 |  |  |  |  |  |
| --- | rs7840990 | 8 | 81584551 | ZNF704 (intronic) | |  | | A/C | 0.03 | 0.07 | 0.36 (0.22-0.58) | 5.22E-06 |  |  |  |  |  |
| --- | rs6473246 | 8 | 81605037 | ZNF704 (intronic) | |  | | T/C | 0.03 | 0.08 | 0.39 (0.25-0.61) | 5.23E-06 |  |  |  |  |  |
| --- | rs9922793 | 16 | 79089885 | WWOX (intronic) | |  | | G/C | 0.05 | 0.03 | 2.12 (1.4-3.21) | 5.23E-06 |  |  |  |  |  |
| --- | rs12102460 | 16 | 79093317 | WWOX (intronic) | |  | | A/G | 0.05 | 0.02 | 2.28 (1.47-3.53) | 5.25E-06 |  |  |  |  |  |
| --- | rs72773698 | 10 | 12869994 | CAMK1D (intronic) | |  | | G/A | 0.38 | 0.47 | 0.69 (0.58-0.82) | 5.26E-06 |  |  |  |  |  |
| --- | rs2881550 | 16 | 79092324 | WWOX (intronic) | |  | | G/A | 0.05 | 0.02 | 2.27 (1.47-3.52) | 5.33E-06 |  |  |  |  |  |
| --- | rs11992435 | 8 | 81526083 |  | | SLC25A51P3,OCIAD2P1,CKS1BP7,RPSAP47,ZNF704,ZBTB10 | | A/G | 0.02 | 0.07 | 0.36 (0.22-0.59) | 5.34E-06 |  |  |  |  |  |
| --- | rs8047303 | 16 | 79087038 | WWOX (intronic) | |  | | C/G | 0.05 | 0.02 | 2.24 (1.47-3.42) | 5.35E-06 |  |  |  |  |  |
| --- | rs73271043 | 8 | 81590171 | ZNF704 (intronic) | |  | | C/T | 0.03 | 0.07 | 0.38 (0.24-0.6) | 5.42E-06 |  |  |  |  |  |
| --- | rs73271047 | 8 | 81591247 | ZNF704 (intronic) | |  | | A/T | 0.03 | 0.07 | 0.38 (0.24-0.61) | 5.45E-06 |  |  |  |  |  |
| --- | rs7833340 | 8 | 81543123 | ZNF704 (utr-variant-3-prime) | |  | | A/T | 0.03 | 0.08 | 0.39 (0.25-0.61) | 5.50E-06 |  |  |  |  |  |
| --- | rs61993625 | 14 | 20546875 |  | | RNA5SP380,OR4K14,OR4L1,OR4Q2,OR4K13,OR4U1P,OR4T1P,OR4K17,OR4N5 | | G/A | 0.16 | 0.11 | 1.56 (1.23-1.98) | 5.50E-06 |  |  |  |  |  |
| --- | rs2167364 | 7 | 50565827 | DDC (intronic) | |  | | T/C | 0.42 | 0.33 | 1.47 (1.24-1.74) | 5.60E-06 |  |  |  |  |  |
| --- | rs12103168 | 16 | 79094319 | WWOX (intronic) | |  | | C/T | 0.05 | 0.02 | 2.28 (1.47-3.53) | 5.62E-06 |  |  |  |  |  |
| --- | rs36093210 | 14 | 20546750 |  | | RNA5SP380,OR4K14,OR4L1,OR4Q2,OR4K13,OR4U1P,OR4T1P,OR4K17,OR4N5 | | T/C | 0.16 | 0.11 | 1.56 (1.23-1.98) | 5.62E-06 |  |  |  |  |  |
| --- | rs10492902 | 16 | 79093873 | WWOX (intronic) | |  | | G/C | 0.05 | 0.02 | 2.27 (1.47-3.52) | 5.62E-06 |  |  |  |  |  |
| --- | rs1037351 | 7 | 50565404 | DDC (intronic) | |  | | T/C | 0.42 | 0.33 | 1.47 (1.24-1.74) | 5.62E-06 |  |  |  |  |  |
| --- | rs2127688 | 8 | 81600304 | ZNF704 (intronic) | |  | | G/A | 0.03 | 0.07 | 0.39 (0.25-0.61) | 5.66E-06 |  |  |  |  |  |
| --- | rs6791694 | 3 | 15947938 |  | | LOC101927674,LOC101927725,IMPDH1P8,ANKRD28,MIR563 | | G/A | 0.25 | 0.18 | 0.66 (0.54-0.8) | 5.74E-06 |  |  |  |  |  |
| --- | rs72846866 | 17 | 77260020 | RBFOX3 (intronic) | |  | | G/A | 0.25 | 0.19 | 1.38 (1.13-1.68) | 5.76E-06 |  |  |  |  |  |
| --- | rs2121732 | 3 | 15954695 |  | | LOC101927674,LOC101927725,IMPDH1P8,ANKRD28,MIR563 | | A/G | 0.26 | 0.18 | 0.66 (0.54-0.8) | 5.76E-06 |  |  |  |  |  |
| --- | rs6992359 | 8 | 81596608 | ZNF704 (intronic) | |  | | T/C | 0.03 | 0.07 | 0.39 (0.25-0.61) | 5.78E-06 |  |  |  |  |  |
| --- | rs12471644 | 2 | 105875798 |  | | LOC100506473,GPR45,LOC644617,C2orf49,TGFBRAP1 | | G/A | 0.09 | 0.06 | 1.65 (1.22-2.24) | 5.78E-06 |  |  |  |  |  |
| --- | rs13330275 | 16 | 79096330 | WWOX (intronic) | |  | | G/C | 0.05 | 0.02 | 2.28 (1.47-3.54) | 5.83E-06 |  |  |  |  |  |
| --- | rs7823175 | 8 | 81605759 | ZNF704 (intronic) | |  | | C/T | 0.03 | 0.07 | 0.39 (0.25-0.61) | 5.88E-06 |  |  |  |  |  |
| --- | rs77441560 | 8 | 81605794 | ZNF704 (intronic) | |  | | A/G | 0.03 | 0.07 | 0.39 (0.25-0.61) | 5.91E-06 |  |  |  |  |  |
| --- | rs113171459 | 8 | 81606258 | ZNF704 (intronic) | |  | | G/T | 0.03 | 0.07 | 0.39 (0.25-0.61) | 5.94E-06 |  |  |  |  |  |
| --- | rs1888170 | 9 | 27629304 |  | | C9orf72,CTAGE12P,MOB3B | | C/G | 0.11 | 0.07 | 1.65 (1.24-2.19) | 6.05E-06 |  |  |  |  |  |
| --- | rs67013610 | 16 | 16860046 |  | |  | | A/G | 0.31 | 0.24 | 1.41 (1.17-1.69) | 6.10E-06 |  |  |  |  |  |
| --- | rs6853829 | 4 | 88811478 |  | | HSP90AB3P,IBSP,MEPE,SPP1 | | C/T | 0.06 | 0.02 | 2.55 (1.68-3.86) | 6.12E-06 |  |  |  |  |  |
| --- | rs67804702 | 16 | 12647861 | SNX29 (intronic) | |  | | T/C | 0.10 | 0.05 | 2 (1.47-2.72) | 6.14E-06 |  |  |  |  |  |
| --- | rs67514565 | 16 | 16858476 |  | |  | | T/C | 0.30 | 0.24 | 1.41 (1.17-1.69) | 6.25E-06 |  |  |  |  |  |
| --- | rs113534464 | 8 | 81597483 | ZNF704 (intronic) | |  | | G/A | 0.03 | 0.07 | 0.39 (0.25-0.61) | 6.26E-06 |  |  |  |  |  |
| --- | rs9923032 | 16 | 79096703 | WWOX (intronic) | |  | | C/T | 0.05 | 0.02 | 2.28 (1.47-3.54) | 6.28E-06 |  |  |  |  |  |
| --- | rs790604 | 6 | 91105349 |  | | MIR4464,BACH2 | | T/C | 0.09 | 0.04 | 2.22 (1.62-3.06) | 6.38E-06 |  |  |  |  |  |
| --- | rs8055606 | 16 | 16874968 |  | |  | | A/G | 0.30 | 0.23 | 1.41 (1.17-1.7) | 6.40E-06 |  |  |  |  |  |
| --- | rs6776325 | 3 | 15956112 |  | | LOC101927674,LOC101927725,IMPDH1P8,ANKRD28,MIR563 | | A/C | 0.25 | 0.18 | 0.66 (0.54-0.8) | 6.42E-06 |  |  |  |  |  |
| --- | rs75708612 | 12 | 3308016 | TSPAN9 (intronic) | |  | | T/C | 0.31 | 0.25 | 1.36 (1.13-1.63) | 6.48E-06 |  |  |  |  |  |
| --- | rs9941999 | 3 | 15958329 |  | | LOC101927674,LOC101927725,IMPDH1P8,ANKRD28,MIR563 | | A/G | 0.25 | 0.18 | 0.66 (0.54-0.8) | 6.54E-06 |  |  |  |  |  |
| --- | rs6806629 | 3 | 140707457 |  | | SLC25A36,RPL23AP41,SPSB4 | | A/G | 0.07 | 0.04 | 0.52 (0.36-0.75) | 6.63E-06 |  |  |  |  |  |
| --- | rs10147729 | 14 | 20545984 |  | | RNA5SP380,OR4K14,OR4L1,OR4Q2,OR4K13,OR4U1P,OR4T1P,OR4K17,OR4N5 | | T/C | 0.16 | 0.11 | 1.56 (1.23-1.98) | 6.65E-06 |  |  |  |  |  |
| --- | rs7647174 | 3 | 15953051 |  | | LOC101927674,LOC101927725,IMPDH1P8,ANKRD28,MIR563 | | T/C | 0.26 | 0.18 | 0.66 (0.54-0.8) | 6.93E-06 |  |  |  |  |  |
| --- | rs73273051 | 8 | 81623146 | ZNF704 (intronic) | |  | | T/C | 0.03 | 0.07 | 0.39 (0.25-0.62) | 6.96E-06 |  |  |  |  |  |
| --- | rs971407 | 20 | 57115118 | APCDD1L-AS1 (intronic) | |  | | G/A | 0.11 | 0.06 | 0.54 (0.41-0.72) | 7.00E-06 |  |  |  |  |  |
| --- | rs73693868 | 8 | 81624693 | ZNF704 (intronic) | |  | | G/A | 0.03 | 0.07 | 0.39 (0.25-0.62) | 7.01E-06 |  |  |  |  |  |
| --- | rs7817889 | 8 | 81631382 | ZNF704 (intronic) | |  | | T/A | 0.03 | 0.07 | 0.39 (0.25-0.62) | 7.08E-06 |  |  |  |  |  |
| --- | rs13264685 | 8 | 81626942 | ZNF704 (intronic) | |  | | T/G | 0.03 | 0.07 | 0.39 (0.25-0.62) | 7.08E-06 |  |  |  |  |  |
| --- | rs2007431 | 8 | 81628005 | ZNF704 (intronic) | |  | | A/G | 0.03 | 0.07 | 0.39 (0.25-0.62) | 7.11E-06 |  |  |  |  |  |
| --- | rs6026339 | 20 | 57111529 | APCDD1L-AS1 (intronic) | |  | | C/G | 0.11 | 0.06 | 0.54 (0.41-0.72) | 7.17E-06 |  |  |  |  |  |
| --- | rs72795632 | 16 | 79090570 | WWOX (intronic) | |  | | A/C | 0.05 | 0.02 | 2.26 (1.46-3.51) | 7.19E-06 |  |  |  |  |  |
| --- | rs35734659 | 8 | 81632757 | ZNF704 (intronic) | |  | | G/C | 0.03 | 0.07 | 0.39 (0.25-0.62) | 7.23E-06 |  |  |  |  |  |
| --- | rs67406414 | 16 | 16860726 |  | |  | | A/G | 0.30 | 0.24 | 1.41 (1.17-1.69) | 7.25E-06 |  |  |  |  |  |
| --- | rs68010225 | 16 | 16860520 |  | |  | | T/C | 0.30 | 0.24 | 1.41 (1.17-1.69) | 7.27E-06 |  |  |  |  |  |
| --- | rs73254812 | 8 | 81634963 | ZNF704 (intronic) | |  | | C/T | 0.03 | 0.07 | 0.39 (0.25-0.62) | 7.29E-06 |  |  |  |  |  |
| --- | rs74618024 | 8 | 81593813 | ZNF704 (intronic) | |  | | C/T | 0.03 | 0.07 | 0.39 (0.25-0.62) | 7.31E-06 |  |  |  |  |  |
| --- | rs7012682 | 8 | 81635920 | ZNF704 (intronic) | |  | | T/G | 0.03 | 0.07 | 0.39 (0.25-0.62) | 7.32E-06 |  |  |  |  |  |
| --- | rs73693874 | 8 | 81636383 | ZNF704 (intronic) | |  | | A/T | 0.03 | 0.07 | 0.4 (0.25-0.62) | 7.34E-06 |  |  |  |  |  |
| --- | rs79649558 | 8 | 81622057 | ZNF704 (intronic) | |  | | G/A | 0.03 | 0.07 | 0.39 (0.25-0.62) | 7.38E-06 |  |  |  |  |  |
| --- | rs74471311 | 8 | 81622847 | ZNF704 (intronic) | |  | | T/C | 0.03 | 0.07 | 0.39 (0.25-0.62) | 7.41E-06 |  |  |  |  |  |
| --- | rs1221559 | 6 | 91110106 |  | | MIR4464 | | G/C | 0.09 | 0.04 | 2.22 (1.61-3.05) | 7.46E-06 |  |  |  |  |  |
| --- | rs58230860 | 17 | 70577564 | LINC00673 (intronic) | |  | | G/A | 0.08 | 0.05 | 1.89 (1.36-2.62) | 7.47E-06 |  |  |  |  |  |
| --- | rs113937511 | 8 | 81625792 | ZNF704 (intronic) | |  | | C/T | 0.03 | 0.07 | 0.39 (0.25-0.62) | 7.48E-06 |  |  |  |  |  |
| --- | rs112635798 | 14 | 60973171 |  | | VN1R59P,C14orf39,SALL4P7,SIX6,RPL37P5,RPS15AP4 | | T/C | 0.05 | 0.02 | 2.11 (1.36-3.28) | 7.49E-06 |  |  |  |  |  |
| --- | rs4685275 | 3 | 15962879 |  | | LOC101927674,LOC101927725,IMPDH1P8,ANKRD28,MIR563 | | G/T | 0.26 | 0.18 | 0.66 (0.54-0.8) | 7.51E-06 |  |  |  |  |  |
| --- | rs148895822 | 3 | 140715172 |  | | SLC25A36,RPL23AP41,SPSB4 | | C/A | 0.05 | 0.02 | 2.36 (1.51-3.71) | 7.51E-06 |  |  |  |  |  |
| --- | rs17472928 | 8 | 81527805 |  | | SLC25A51P3,OCIAD2P1,CKS1BP7,RPSAP47,ZNF704,ZBTB10 | | A/G | 0.03 | 0.07 | 0.37 (0.23-0.6) | 7.53E-06 |  |  |  |  |  |
| --- | rs2345743 | 3 | 15945191 |  | | LOC101927674,LOC101927725,IMPDH1P8,ANKRD28,MIR563 | | C/T | 0.26 | 0.19 | 0.66 (0.54-0.81) | 7.54E-06 |  |  |  |  |  |
| --- | rs4685276 | 3 | 15962985 |  | | LOC101927674,LOC101927725,IMPDH1P8,ANKRD28,MIR563 | | T/C | 0.26 | 0.18 | 0.66 (0.54-0.8) | 7.54E-06 |  |  |  |  |  |
| --- | rs9866128 | 3 | 15966433 |  | | LOC101927674,LOC101927725,IMPDH1P8,ANKRD28,MIR563 | | T/G | 0.25 | 0.18 | 0.66 (0.54-0.81) | 7.56E-06 |  |  |  |  |  |
| --- | rs73514347 | 16 | 16860874 |  | |  | | G/A | 0.30 | 0.24 | 1.41 (1.17-1.69) | 7.60E-06 |  |  |  |  |  |
| --- | rs59376819 | 8 | 81638954 | ZNF704 (intronic) | |  | | G/A | 0.03 | 0.07 | 0.4 (0.25-0.62) | 7.60E-06 |  |  |  |  |  |
| --- | rs2161726 | 16 | 79091497 | WWOX (intronic) | |  | | G/T | 0.05 | 0.02 | 2.24 (1.45-3.46) | 7.62E-06 |  |  |  |  |  |
| --- | rs73260919 | 8 | 81525926 |  | | SLC25A51P3,OCIAD2P1,CKS1BP7,RPSAP47,ZNF704,ZBTB10 | | G/A | 0.02 | 0.07 | 0.37 (0.22-0.6) | 7.69E-06 |  |  |  |  |  |
| --- | rs2923178 | 5 | 165930935 |  | |  | | G/A | 0.32 | 0.24 | 0.67 (0.56-0.81) | 7.72E-06 |  |  |  |  |  |
| --- | rs1002371 | 7 | 50488209 |  | | IKZF1,DDC,FIGNL1 | | C/T | 0.24 | 0.16 | 1.67 (1.36-2.05) | 7.76E-06 |  |  |  |  |  |
| --- | rs1221554 | 6 | 91115991 |  | | MIR4464 | | A/G | 0.09 | 0.04 | 2.22 (1.61-3.05) | 7.81E-06 |  |  |  |  |  |
| --- | rs73254821 | 8 | 81641392 | ZNF704 (intronic) | |  | | A/G | 0.03 | 0.07 | 0.4 (0.25-0.62) | 7.86E-06 |  |  |  |  |  |
| --- | rs28573146 | 7 | 50485589 |  | | IKZF1,DDC,FIGNL1 | | C/T | 0.24 | 0.16 | 1.67 (1.36-2.04) | 7.89E-06 |  |  |  |  |  |
| rs6992620 | rs6992620 | 8 | 81635971 | ZNF704 (intronic) | |  | | C/T | 0.03 | 0.07 | 0.4 (0.25-0.62) | 7.95E-06 |  |  |  |  |  |
| --- | rs73693881 | 8 | 81643240 | ZNF704 (intronic) | |  | | T/C | 0.03 | 0.07 | 0.4 (0.25-0.63) | 8.07E-06 |  |  |  |  |  |
| --- | rs4685277 | 3 | 15963163 |  | | LOC101927674,LOC101927725,IMPDH1P8,ANKRD28,MIR563 | | T/C | 0.26 | 0.18 | 0.66 (0.54-0.8) | 8.09E-06 |  |  |  |  |  |
| --- | rs12103013 | 16 | 79093502 | WWOX (intronic) | |  | | C/G | 0.05 | 0.02 | 2.25 (1.45-3.49) | 8.41E-06 |  |  |  |  |  |
| --- | rs4685270 | 3 | 15942480 |  | | LOC101927674,LOC101927725,IMPDH1P8,ANKRD28,MIR563 | | C/T | 0.28 | 0.20 | 0.67 (0.55-0.81) | 8.42E-06 |  |  |  |  |  |
| --- | rs11779571 | 8 | 18544452 | PSD3 (intronic) | |  | | T/C | 0.56 | 0.47 | 0.7 (0.59-0.83) | 8.43E-06 |  |  |  |  |  |
| --- | rs112362062 | 8 | 81648661 | ZNF704 (intronic) | |  | | C/T | 0.03 | 0.07 | 0.4 (0.26-0.63) | 8.48E-06 |  |  |  |  |  |
| --- | rs111897958 | 8 | 81641637 | ZNF704 (intronic) | |  | | G/A | 0.03 | 0.07 | 0.4 (0.25-0.63) | 8.51E-06 |  |  |  |  |  |
| --- | rs9870942 | 3 | 15967128 |  | | LOC101927674,LOC101927725,IMPDH1P8,ANKRD28,MIR563 | | T/G | 0.26 | 0.18 | 0.66 (0.54-0.8) | 8.69E-06 |  |  |  |  |  |
| --- | rs6442553 | 3 | 15927193 |  | | LOC101927674,LOC101927725,IMPDH1P8,ANKRD28,MIR563 | | A/G | 0.27 | 0.20 | 0.67 (0.55-0.81) | 8.70E-06 |  |  |  |  |  |
| --- | rs112379021 | 8 | 81643195 | ZNF704 (intronic) | |  | | T/G | 0.03 | 0.07 | 0.4 (0.26-0.63) | 8.75E-06 |  |  |  |  |  |
| --- | rs113139265 | 8 | 81643928 | ZNF704 (intronic) | |  | | C/T | 0.03 | 0.07 | 0.4 (0.26-0.63) | 8.83E-06 |  |  |  |  |  |
| --- | rs1447645 | 3 | 15930342 |  | | LOC101927674,LOC101927725,IMPDH1P8,ANKRD28,MIR563 | | T/C | 0.27 | 0.20 | 0.67 (0.55-0.81) | 8.85E-06 |  |  |  |  |  |
| --- | rs4685271 | 3 | 15948462 |  | | LOC101927674,LOC101927725,IMPDH1P8,ANKRD28,MIR563 | | T/C | 0.25 | 0.18 | 0.66 (0.54-0.81) | 8.90E-06 |  |  |  |  |  |
| --- | rs1447647 | 3 | 15933465 |  | | LOC101927674,LOC101927725,IMPDH1P8,ANKRD28,MIR563 | | G/A | 0.27 | 0.20 | 0.67 (0.55-0.81) | 8.99E-06 |  |  |  |  |  |
| --- | rs2084355 | 3 | 15935331 |  | | LOC101927674,LOC101927725,IMPDH1P8,ANKRD28,MIR563 | | G/A | 0.27 | 0.20 | 0.67 (0.55-0.81) | 9.08E-06 |  |  |  |  |  |
| --- | rs9835592 | 3 | 15936942 |  | | LOC101927674,LOC101927725,IMPDH1P8,ANKRD28,MIR563 | | A/G | 0.27 | 0.20 | 0.67 (0.55-0.81) | 9.17E-06 |  |  |  |  |  |
| --- | rs1345158 | 3 | 15931704 |  | | LOC101927674,LOC101927725,IMPDH1P8,ANKRD28,MIR563 | | G/T | 0.27 | 0.20 | 0.67 (0.55-0.81) | 9.18E-06 |  |  |  |  |  |
| --- | rs3846131 | 3 | 15932357 |  | | LOC101927674,LOC101927725,IMPDH1P8,ANKRD28,MIR563 | | C/T | 0.27 | 0.20 | 0.67 (0.55-0.81) | 9.21E-06 |  |  |  |  |  |
| --- | rs1579901 | 3 | 15938463 |  | | LOC101927674,LOC101927725,IMPDH1P8,ANKRD28,MIR563 | | C/G | 0.27 | 0.20 | 0.67 (0.55-0.81) | 9.24E-06 |  |  |  |  |  |
| --- | rs3843387 | 3 | 15938909 |  | | LOC101927674,LOC101927725,IMPDH1P8,ANKRD28,MIR563 | | C/A | 0.27 | 0.20 | 0.67 (0.55-0.81) | 9.25E-06 |  |  |  |  |  |
| --- | rs141587987 | 7 | 99938483 | PILRB (intronic) | |  | | C/T | 0.05 | 0.02 | 2.16 (1.38-3.36) | 9.25E-06 |  |  |  |  |  |
| --- | rs6791487 | 3 | 15947721 |  | | LOC101927674,LOC101927725,IMPDH1P8,ANKRD28,MIR563 | | G/A | 0.26 | 0.19 | 0.66 (0.54-0.81) | 9.25E-06 |  |  |  |  |  |
| --- | rs1466072 | 3 | 15939311 |  | | LOC101927674,LOC101927725,IMPDH1P8,ANKRD28,MIR563 | | G/T | 0.27 | 0.20 | 0.67 (0.55-0.81) | 9.27E-06 |  |  |  |  |  |
| --- | rs2345742 | 3 | 15939832 |  | | LOC101927674,LOC101927725,IMPDH1P8,ANKRD28,MIR563 | | C/T | 0.27 | 0.20 | 0.67 (0.55-0.81) | 9.44E-06 |  |  |  |  |  |
| --- | rs79549559 | 8 | 81652338 | ZNF704 (intronic) | |  | | A/C | 0.03 | 0.07 | 0.41 (0.26-0.63) | 9.45E-06 |  |  |  |  |  |
| --- | rs4593007 | 3 | 15952252 |  | | LOC101927674,LOC101927725,IMPDH1P8,ANKRD28,MIR563 | | A/T | 0.26 | 0.19 | 0.67 (0.55-0.81) | 9.47E-06 |  |  |  |  |  |
| --- | rs2881211 | 3 | 15866983 | ANKRD28 (intronic) | |  | | C/T | 0.29 | 0.21 | 0.67 (0.56-0.81) | 9.48E-06 |  |  |  |  |  |
| --- | rs74396987 | 8 | 81652600 | ZNF704 (intronic) | |  | | G/A | 0.03 | 0.07 | 0.41 (0.26-0.63) | 9.48E-06 |  |  |  |  |  |
| --- | rs6442549 | 3 | 15805224 | ANKRD28 (intronic) | |  | | A/G | 0.29 | 0.21 | 0.67 (0.56-0.81) | 9.53E-06 |  |  |  |  |  |
| --- | rs13059670 | 3 | 15966961 |  | | LOC101927674,LOC101927725,IMPDH1P8,ANKRD28,MIR563 | | G/C | 0.26 | 0.19 | 0.66 (0.54-0.8) | 9.61E-06 |  |  |  |  |  |
| --- | rs111314634 | 8 | 81648989 | ZNF704 (intronic) | |  | | T/C | 0.03 | 0.07 | 0.4 (0.26-0.63) | 9.69E-06 |  |  |  |  |  |
| --- | rs9852108 | 3 | 15967650 |  | | LOC101927674,LOC101927725,IMPDH1P8,ANKRD28,MIR563 | | G/A | 0.26 | 0.19 | 0.66 (0.54-0.8) | 9.70E-06 |  |  |  |  |  |
| --- | rs9877368 | 3 | 15958003 |  | | LOC101927674,LOC101927725,IMPDH1P8,ANKRD28,MIR563 | | T/C | 0.26 | 0.19 | 0.67 (0.55-0.82) | 9.71E-06 |  |  |  |  |  |
| rs1466073 | rs1466073 | 3 | 15940832 |  | | LOC101927674,LOC101927725,IMPDH1P8,ANKRD28,MIR563 | | A/G | 0.27 | 0.20 | 0.67 (0.55-0.81) | 9.78E-06 |  |  |  |  |  |
| --- | rs145651160 | 8 | 17926303 | ASAH1 (intronic) | |  | | C/T | 0.06 | 0.02 | 2.36 (1.57-3.55) | 9.85E-06 |  |  |  |  |  |
| --- | rs6995287 | 8 | 81585160 | ZNF704 (intronic) | |  | | T/G | 0.03 | 0.07 | 0.39 (0.25-0.62) | 9.90E-06 |  |  |  |  |  |
| Abbreviations: MAF, Minor Allele Frequency; Chr, Chromosome; CI, Confidence Interval | | | | | | | | | | | | |  |  |  |  |  |
| ^a^ Genotyped SNPs indicated in this column, dashed entries indicate imputed SNPs. ^b^ Allele B is the test allele for all SNPs, but is not necessarily the minor allele. ^c^ Odds ratios are B versus the A allele. | | | | | | | | | | | | |  |  |  |  |  |

**Supplementary Table S3: Chromosome 8 SNPs with p-values suggestive of association with childhood ALL.** Replication using a French cohort [[Orsi et al., 2012](#_ENREF_7_1)] was attempted but not successful for three SNPs proximal to *ZNF704* which had been validated using TaqMan assays.

|  |  |  |  |  | MAF | | ALL vs Controls -Australian | | ALL vs Controls-French | |
| --- | --- | --- | --- | --- | --- | --- | --- | --- | --- | --- |
| SNP | Cytoband | Position (b37) | Genotype (A/B)^a^ | Gene | ALL (n=358) *[French replication]* | Controls (1192) *[French replication]* | Allelic OR (95% confidence interval) | P | Allelic OR (95% confidence interval) | P |
| rs7000234 | 8q21.13 | 81537114 | G/A | *~ZNF704* | 0.03 *[Absent]* | 0.07 *[Absent]* | 0.35 (0.22-0.57) | 2.62E-06 | NA | NA |
| rs7018449 | 8q21.13 | 81576965 | T/C | *ZNF704* | 0.03 *[0.07]* | 0.07 *[0.07]* | 0.36 (0.22-0.57) | 3.24E-06 | 1.00 (0.75-1.33) | 0.99 |
| rs6992620 | 8q21.13 | 81635971 | C/T | *~ZNF704* | 0.03 *[0.08]* | 0.07 *[0.08]* | 0.40 (0.25-0.62) | 7.95E-06 | 1.01 (0.70-1.03) | 0.97 |

**^a^** Allele B is the minor allele.

Orsi L, Rudant J, Bonaventure A, Goujon-Bellec S, Corda E, Evans TJ, Petit A, Bertrand Y, Nelken B, Robert A, Michel G, Sirvent N, Chastagner P, Ducassou S, Rialland X, Hémon D, Milne E, Scott RJ, Baruchel A & Clavel J 2012. Genetic polymorphisms and childhood acute lymphoblastic leukemia: GWAS of the ESCALE study (SFCE). Leukemia 26**,** 2561-4.

**Supplementary Table S4: Estimates for genetic main effects of variation at rs7089424 and interaction with parental exposures, age, and sex for 204 case-trios.**

| Variable Name | Alleles | P | Environmental variable present | Genotype-wise Association | | | |
| --- | --- | --- | --- | --- | --- | --- | --- |
|  |  |  |  | **Cases: Controls** ^a^ | **OR** ^b^ | **95% CI** | **P value** |
| rs7089424 | GG | 0.09 ^c^ |  | 44:107 | 1.88 | 1.06-3.36 | 0.03 |
|  | GT |  |  | 99:287 | 1.42 | 0.92-2.18 | 0.11 |
|  | TT (REF) |  |  | 62:221 | 1 |  |  |
| Mother alcohol use before pregnancy | | | | | | | |
|  | GG | 0.34 ^d^ | Yes | 31:71 | 1.77 | 0.85-3.62 | 0.13 |
|  | GT |  |  | 61:95 | 1.13 | 0.66-1.95 | 0.66 |
|  | TT |  |  | 41:133 | 1 |  |  |
|  | GG |  | No | 13:36 | 2.03 | 0.76-5.42 | 0.16 |
|  | GT |  |  | 38:92 | 2.01 | 0.99-4.06 | 0.05 |
|  | TT |  |  | 21:88 | 1 |  |  |
| Mother folic acid use before pregnancy | | | | | | | |
|  | GG | 0.36 ^d^ | Yes | 23:48 | 2.83 | 1.04-7.69 | 0.04 |
|  | GT |  |  | 28:92 | 1.47 | 0.65-3.30 | 0.35 |
|  | TT |  |  | 19:70 | 1 |  |  |
|  | GG |  | No | 20:58 | 1.43 | 0.69-2.99 | 0.34 |
|  | GT |  |  | 71:191 | 1.45 | 0.88-2.39 | 0.15 |
|  | TT |  |  | 42:150 | 1 |  |  |
| Father smoked during 2 years before pregnancy | | | | | | | |
|  | GG | 0.79 ^d^ | Yes | 25:66 | 2.50 | 0.79-7.89 | 0.12 |
|  | GT |  |  | 64:178 | 2.00 | 0.87-4.59 | 0.10 |
|  | TT |  |  | 33:122 | 1 |  |  |
|  | GG |  | No | 13:33 | 1.66 | 0.80-3.42 | 0.17 |
|  | GT |  |  | 32:84 | 1.45 | 0.85-2.48 | 0.17 |
|  | TT |  |  | 23:87 | 1 |  |  |
| Patient sex | | | | | | | |
|  | GG | 0.96 ^d^ | Male | 27:67 | 2.01 | 0.90-4.50 | 0.09 |
|  | GT |  |  | 56:162 | 1.52 | 0.82-2.84 | 0.19 |
|  | TT |  |  | 27:101 | 1 |  |  |
|  | GG |  | Female | 17:40 | 1.78 | 0.77-4.11 | 0.18 |
|  | GT |  |  | 43:125 | 1.33 | 0.74-2.40 | 0.34 |
|  | TT |  |  | 35:120 | 1 |  |  |
| Patient age |  |  | **Mean Age (Cases:Controls)** | | **OR** | **95% CI** | **P** |
|  | GG | 0.72 ^d^ |  | 4.8 : 5.0 | 0.94 | 0.80-1.10 | 0.42 |
|  | GT |  |  | 5.2 : 5.2 | 0.97 | 0.86-1.09 | 0.58 |
|  | TT |  |  | 5.8 : 5.5 | 1 |  |  |
| \| Abbreviations: OR, Odds Ratio; CI, Confidence Interval; P, P-value.  ^a^ Case numbers reduced from GWAS according to the availability of both, parent genotypes and completed questionnaire; three matched controls per case were theoretically deduced from known parent genotypes. ^b^ Referent group was genotype TT for all ORs. ^c^ likelihood ratio test p-value for main genetic effect (2df). ^d^ likelihood ratio test p-value for additional interaction (2df) comparing environmental exposure categories. \| \| --- \| | | | | | | | |
